# Factors associated with long-term benzodiazepine and Z-drug use across the lifespan and 5-year temporal trajectories among incident users: a Swedish nationwide register-based study

**DOI:** 10.1007/s00228-023-03515-2

**Published:** 2023-06-09

**Authors:** Kayoko Isomura, Xinchen Wang, Zheng Chang, Clara Hellner, Jan Hasselström, Isabella Ekheden, Nitya Jayaram-Lindström, Paul Lichtenstein, Brian M. D’Onofrio, David Mataix-Cols, Anna Sidorchuk

**Affiliations:** 1 Centre for Psychiatry Research, Department of Clinical Neuroscience, Karolinska Institutet, Stockholm, Sweden; 2 Stockholm Health Care Services, Region Stockholm, Stockholm, Sweden; 3 Department of Medical Epidemiology and Biostatistics, Karolinska Institutet, Stockholm, Sweden; 4 Department of Neurobiology, Care Sciences and Society, Karolinska Institutet, Stockholm, Sweden; 5 Academic Primary Health Care Centre, Region Stockholm, Stockholm, Sweden; 6 Division of Clinical Pharmacology, Department of Laboratory Medicine, Karolinska Institutet, Stockholm, Sweden; 7 Department of Psychological and Brain Sciences, Indiana University, Bloomington, USA

**Keywords:** Benzodiazepines, Z-drugs, Long-term use, Prescription, Dispensation, Trajectory

## Abstract

**Purpose:**

Despite being discouraged by guidelines, long-term use of benzodiazepines and related Z-drugs (BZDR) remains frequent in the real-world. An improved understanding of factors associated with the transition from new to long-term BZDR use and of temporal BZDR use trajectories is needed. We aimed to assess the proportion of long-term BZDR use (> 6 months) in incident BZDR-recipients across the lifespan; identify 5-year BZDR use trajectories; and explore individual characteristics (demographic, socioeconomic and clinical) and prescribing-related factors (pharmacological properties of the initial BZDR, prescriber’s healthcare level, and concurrent dispensing of other medications) associated with long-term BZDR use and distinct trajectories.

**Methods:**

Our nationwide register-based cohort included all BZDR-recipients in Sweden with first dispensation in 2007–2013. Trajectories of BZDR use days per year were built using group-based trajectory modelling. Cox regression and multinomial logistic regression were fitted to assess the predictors of long-term BZDR use and trajectories’ membership.

**Results:**

In 930,465 incident BZDR-recipients, long-term use increased with age (20.7%, 41.0%, and 57.4% in 0–17, 18–64, and ≥ 65-year-olds, respectively). Four BZDR use trajectories emerged, labelled ‘discontinued’, ‘decreasing’, ‘slow decreasing’ and ‘maintained’. The proportion of the ‘discontinued’ trajectory members was the largest in all ages, but reduced from 75.0% in the youths to 39.3% in the elderly, whereas the ‘maintained’ increased with age from 4.6% to 36.7%. Prescribing-related factors, in particular multiple BZDRs at initiation and concurrent dispensing of other medications, were associated with increased risks of long-term (*vs* short-term) BZDR use and developing other trajectories (*vs* ‘discontinued’) in all age groups.

**Conclusions:**

The findings highlight the importance of raising awareness and providing support to prescribers to make evidence-based decisions on initiating and monitoring BZDR treatment across the lifespan.

**Supplementary Information:**

The online version contains supplementary material available at 10.1007/s00228-023-03515-2.

## Introduction

A broad spectrum of pharmacological properties and rapid onset of therapeutic effects placed benzodiazepines (BZD) and related Z-drugs (henceforth ‘BZDR’ if BZDs and Z-drugs are jointly mentioned) among the most widely used psychotropic medications worldwide [1]. BZDs are mainly indicated for alleviating anxiety and insomnia symptoms (the latter being the main indication also for Z-drugs), and managing alcohol withdrawal in adults [2–4], and for treating epilepsy or seizure disorders in all ages [5]. Clinical guidelines from Sweden and other countries recommend the use of BZDRs at the lowest effective dose and for the shortest possible duration of treatment (i.e., of a few consecutive weeks) [6–11], owing to the risks of developing tolerance, physiologic and psychological dependence, and severe adverse effects, including psychomotor impairment and cognitive decline [12–15], as well as due to limited amount of evidence of BZDR long-term effectiveness and safety [16, 17]. Over last decades, an array of guidelines and disease-specific recommendations have been issued in the Nordic countries making the regulations about the use of BZDRs more stringent towards specific clinical indications and for shorter duration of treatment [18]. Despite the concerns on the risks of long-term BZDR use, continuous prescribing extending for months or even years is frequently reported [19], mainly in studies on elderly patients (ranging 12–54% among the recipients aged ≥ 65 years [20–22]), but also in children and adolescents (3–31% in patients < 18 years [23–27]), and among adults (9–34% in 18–64-year-olds [22, 28, 29]), with variations in proportions depending on study definition of long-term use, prevalent or incident data, country, and the studied drugs. Although, there is no standard definition of long-term BZDR use, it is commonly defined as the use for a period longer than 6 months [19]. While a prolonged treatment might be considered justified for some clinical situations (e.g., failure to respond to other treatments, palliative care, some seizure disorders) [28, 30, 31], these represent a minority of patients, beyond which long-term BZDR use remains a controversial clinical practice and generates professional debates [31–34].

To gain insights into a possible discrepancy between clinical recommendations and the existing BZDR prescribing practices, it may be helpful to focus on factors underlying the transition from new (i.e., incident) to long-term BZDR prescribing. Prior studies on the incident BZDR-recipients reported several factors that might predict long-term prescribing, including older age, somatic and psychiatric comorbidities, types of the initial drug(s), healthcare level where treatment was initiated, although, the reported factors varied between studies [20, 22, 29, 35–39]. Better knowledge on driving forces of long-term prescribing in different patient groups is needed to optimize the prescribing practices; yet, the existing evidence remains scarce and mainly comes from studies on adults with a rare focus on children and adolescents [23].

Another area that requires further study is the temporal trajectory of BZDR use because it is likely to be dynamic over time. Indeed, two French studies on BZDR prescribing frequency revealed that in addition to a short-term (occasional) use, chronic users of BZD-anxiolytics [40] and BZD-hypnotics and Z-drugs [41] followed different temporal trajectories (e.g., early increasing, late increasing, increasing/decreasing, quasi-continues use). Similar heterogeneity in trajectories of chronic BZDR use were shown in studies from Taiwan [27] and Canada [42]. Overall, this emerging literature suggested that different use trajectories may be associated with different patient characteristics and possible barriers to reducing or stopping BZDRs. This work emphasizes the importance of improving our knowledge on BZDR use trajectories and their predictors to guide clinicians to reduce the risk of harmful BZDR prescribing [27, 40–42].

In this study, we leveraged Swedish nationwide registers to 1) describe the proportion of long-term BZDR users (with data on dispensation as a proxy for use) among incident BZDR-recipients from different age groups (childhood and adolescence, adulthood, old age); 2) identify BZDR use trajectories among the participants who remained under study for at least 5 years after the first BZDR dispensation; and 3) assess individual characteristics (demographic, socioeconomic and clinical) and prescribing-related factors (pharmacological properties of the initial BZDR, prescriber’s healthcare level, and concurrent dispensing of other medications) associated with the risk of developing long-term BZDR use and with the membership in distinct trajectories.

## Methods

### Study population

Data were retrieved from the Swedish nationwide registers (see Supplementary Note 1) and linked via the unique identification number assigned to all Swedish residents [43]. Information on BZDR dispensations was obtained from the Prescribed Drug Register (PDR) [44] and based on the Anatomical Therapeutic Chemical (ATC) codes for benzodiazepine derivatives in anxiolytics (N05BA), hypnotics/sedatives (N05CD), antiepileptics (N03AE), and Z-drugs (N05CF) (Supplementary Table S1).

**Fig. 1:**
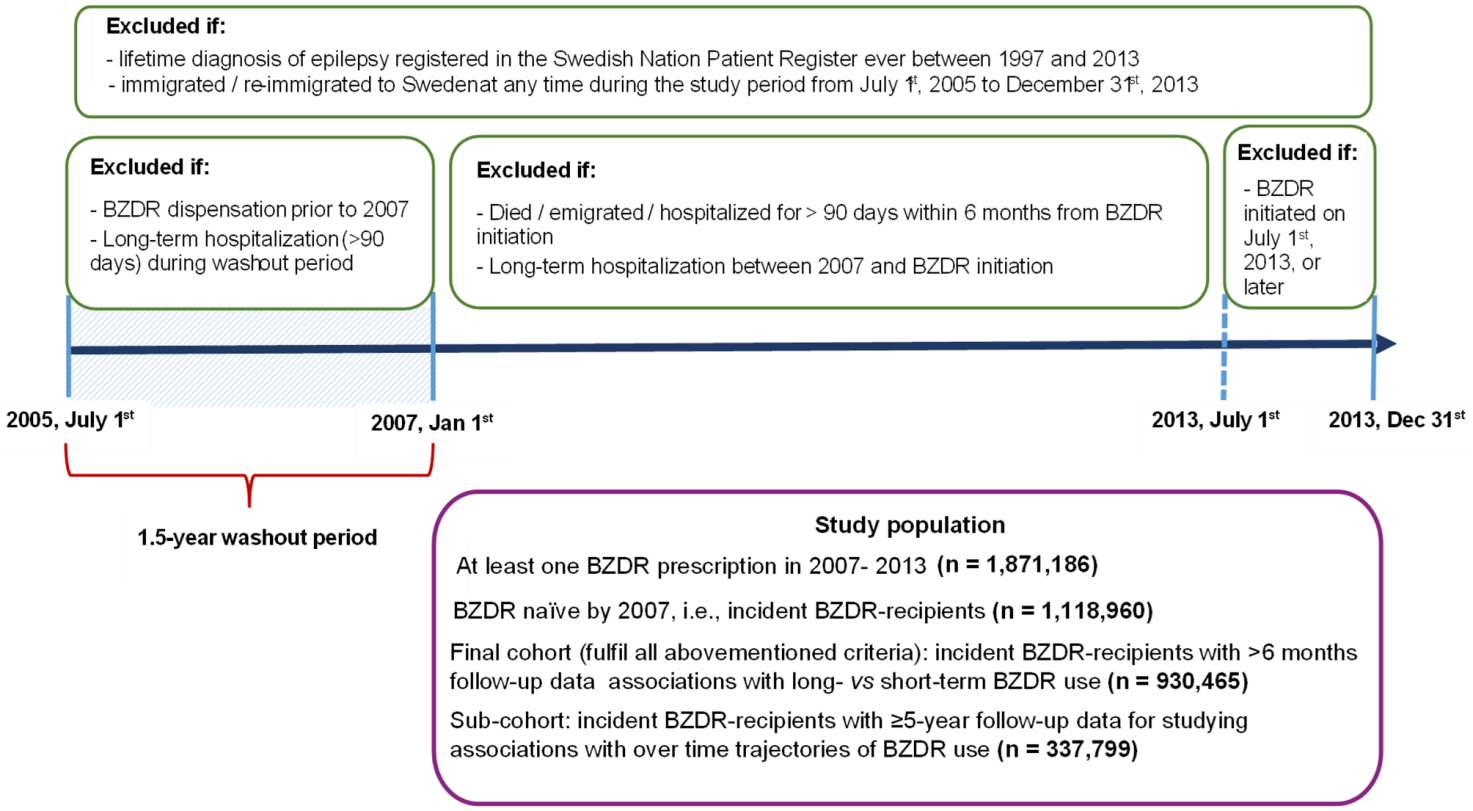
Study cohort flow-chart

Among individuals who were dispensed at least one BZDR prescription in 2007–2013 (n = 1,871,186), we selected incident recipients (n = 1,118,960) (Fig. 1). We excluded individuals if they: 1) were dispensed any BZDR before January 1^st^, 2007 (i.e., during a 1.5-year washout period from the PDR inception on July 1^st^, 2005); 2) were hospitalized for longer than 90 days (according to the National Patient Register) during a washout period or ever before the first BZDR dispensation; and 3) immigrated or re-immigrated to Sweden after July 1^st^, 2005 (as recorded in the Total Population Register). Further, to distinguish between short-term and long-term BZDR use, we excluded BZDR-recipients who died (according to the Cause of Death Register), emigrated, or were hospitalized for > 90 days within 6 months after the first dispensation, or if BZDR was dispensed for the first time on July 1^st^, 2013, or later (i.e., within less than 6 months before the study end on December 31^st^, 2013). Finally, we excluded individuals with a lifetime history of epilepsy since a prolonged medication use can be indicated for this condition. Following the exclusions, the final cohort consisted of 930,465 incident BZDR-recipients (58.8% women) and was then divided into three groups, based on the age of the first dispensation: 0–17 years (n = 18,484, 51.2% women), 18–64 years (n = 590,720, 58.9% women), and ≥ 65 years (n = 321,261, 58.9% women).

### BZDR use measures

Register data on BZDR dispensation were employed as a proxy for BZDR use. To explore different patterns of BZDR use, first, we constructed a dichotomous measure of *long-term vs short-term BZDR use*. For this, in line with prior research [26, 45], for each participant we retrieved dates of each dispensation of any BZDR during the follow-up and defined an ‘individual treatment period’ as a sequence of BZDR dispensations if the gap between two consecutive dispensation dates, did not exceed 6 months (regardless of whether the same or different BZDRs were dispensed each time). The duration of each individual treatment period was estimated as the length of time (i.e., the number of days) between the first and final dispensations. With a gap extending beyond 6 months, the next dispensation was considered as the initiation of a new individual treatment period. As the Swedish Pharmaceutical Benefits allow for a maximum of 3-month medication supply per prescription [46], the duration of each period was then extended by 91 days to capture the full length of BZDR treatment. Thus, during the follow-up, each person could have had more than one treatment period of various durations, with a minimal length of a single individual treatment period of 3 months. Those with at least one period longer than 6 months [19] were defined as having ‘long-term BZDR use’, otherwise, as having a ‘short-term use’.

**Fig. 2:**
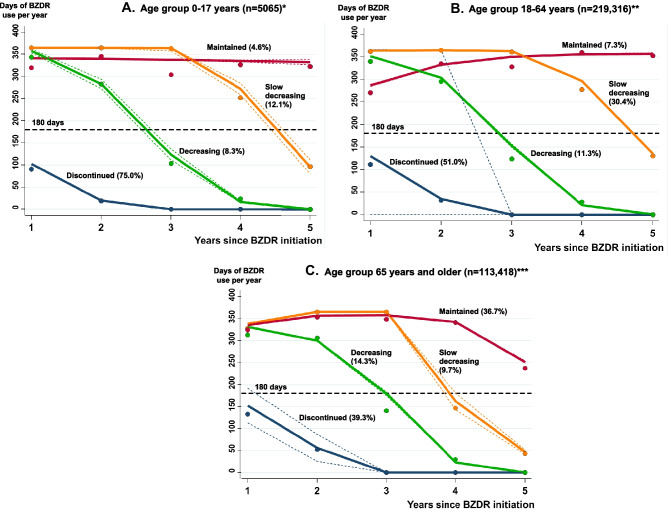
The trajectory models of predicted probability of BZDR use (solid lines) with 95% confidence intervals (short-dashed lines) over 5-year period within the age group of 0–17 years at the treatment initiation (**A**), 18–64 years (**B**), and 65 years and older (**C**), built by group-based trajectory modelling. *Note*: Proportions reported next to each trajectory name denote the proportions of BZDR-recipients assigned to each trajectory-group (i.e., observed values). * For 0–17 year age group, the number of BZDR-recipients assigned to each trajectory-group and the corresponding proportions (in parentheses) as well as the proportions of estimated probability of group membership, i.e., predicted values [in brackets] are as the following: the ‘Discontinued’ trajectory n = 3798 (75.0%) [74.5%], the ‘Decreasing’ trajectory n = 420 (8.3%) [8.8%], the ‘Slow decreasing’ trajectory n = 614 (12.1%) [11.9], and the ‘Maintained’ trajectory n = 233 (4.6%) [4.8%]. ** For 18–64 year age group, the number of BZDR-recipients assigned to each trajectory-group and the corresponding proportions (in parentheses) as well as the proportions of estimated probability of group membership, i.e., predicted values [in brackets] are as the following: the ‘Discontinued’ n = 111,959 (51.0%) [50.5%], the ‘Decreasing’ n = 24,707 (11.3%) [12.1%], the ‘Slow decreasing’ n = 66,625 (30.4%) [29.6%], and the ‘Maintained’ n = 16,025 (7.3%) [7.8%]. *** For the age group of ≥ 65 years, the number of BZDR-recipients assigned to each trajectory-group and the corresponding proportions (in parentheses) as well as the proportions of estimated probability of group membership, i.e., predicted values [in brackets] are as the following: the ‘Discontinued’ n = 44,525 (39.3%) [38.5%], the ‘Decreasing’ n = 16,220 (14.3%) [15.5%], the ‘Slow decreasing’ n = 11,014 (9.7%) [9.9%], and the ‘Maintained’ n = 41,659 (36.7%) [36.2%]

Second, we constructed *trajectories of BZDR use* for the participants who had at least 5 years of follow-up after BZDR initiation (i.e., who were dispensed the first BZDR not later than on December 31^st^, 2008, and who did not die, emigrate, or have > 90 days hospitalization during 5 years after BZDR initiation). Using the same approach as described above for constructing the individual treatment periods, for each person, we estimated the number of BZDR use days, starting from the day of BZDR treatment initiation, as the sum of days of all individual treatment periods per year within each of 5 consecutive years. Thus, within each separate year under a 5-year follow-up, each study participant could have between 0 days to up to 365 days of estimated BZDR use based on the number of individual treatment periods and the length (in days) of such periods defined within a year. Then, in each age category, we applied the group-based trajectory modelling (GBTM) [47, 48] to establish the groups that followed distinct trajectories regarding the number of BZDR use days per year. The GBTM assigned individuals to the group (not pre-defined) for which they showed statistically similar developmental course. For all ages, the best fitting models were identified by the GBTM as 4-group trajectories (see Supplementary Note 2). Figure 2 visualises the trajectories, which were named: 1) ‘*Discontinued*’, if BZDRs were dispensed for only one or two consecutive years after initiation; 2) ‘*Decreasing*’, if the number of BZDR use days were constantly declining until reaching zero; 3) ‘*Slow decreasing*’, if the number of BZDR use days were declining more gradually with a period of no change at the beginning of follow-up; and 4) ‘*Maintained*’, if the number of BZDR use days remained high and relatively stable across the follow-up.

### Covariates

We collected data on several prescribing-related factors and individual characteristics, which in prior research were suggested to be associated with long-term BZDR use [22, 23, 36, 37, 49]. Information on prescribing-related factors was obtained from the PDR. We categorised the initial BZDRs by its pharmacological properties into BZD-anxiolytics, BZD-hypnotics/sedatives, BZD-antiepileptics, Z-drugs, and ‘multiple BZDRs at initiation’, if the initial prescription contained more than one BZDR drug (see Supplementary Table S3 footnotes for details) [50]. Then, we retrieved information on prescriber’s healthcare level where the first BZDR was prescribed as primary care, specialized non-psychiatric care, psychiatric care, and ‘multiple prescribers’ (if patient simultaneously filled in several prescriptions issued at different healthcare services). Also, we collected data on concurrent dispensations of other psychotropic, antiepileptic, and analgesic medications, if such medications were dispensed within 3 months prior to BZDR initiation (Supplementary Table S1). To gain information on individuals characteristics, from the Total Population Register we retrieved demographic data on individual’s sex and country of birth, and from the Small Areas for Market Statistics Register we collected information on residence in Swedish counties at the time of BZDR initiation. Also, from the Longitudinal Integration Database for Health Insurance and Labour Market Studies we collected socioeconomic data on civil status, disposable income (as a proxy for socioeconomic status), unemployment, disability pension, and social welfare recorded during the year before BZDR initiation (for individuals aged 0–17, socioeconomic data were collected separately for their mothers and fathers, who were linked to the study participants by means of the Multi-Generation Register, while for ≥ 65-year-olds, unemployment, disability pension, and social welfare data were not applicable). In addition, we retrieved clinical data on the history of psychiatric and somatic disorders, if recorded between 1997 (when the International Classification of Diseases, Tenth Edition [ICD-10] was introduced) and the first BZDR dispensation (Supplementary Table S2).

### Statistical analysis

For descriptive purposes, within each age group we presented the distribution of BZDR-recipients with long-term use (dichotomously constructed) by the type of the initial BZDR as well as the proportions of those who progressed to long-term use immediately at initiation (i.e., if the first treatment period was > 6 months). Next, Cox proportional hazards regression models were fitted to assess the risk of developing long-term BZDR use among participants with different individual and prescribing-related characteristics. Participants were followed from the date of the first BZDR dispensation until the date they fulfilled the definition for long-term use (i.e., then the individual treatment period reached the length of > 6 months [19, 26]), death, emigration, the admission date for > 90 days hospitalization, or the study end on December 31^st^, 2013, whichever occurred first.

In a sub-cohort of BZDR-recipients with at least 5-year follow-up, we fitted multinomial logistic regression models to compare the odds of belonging to the ‘decreasing’, ‘slow decreasing’, and ‘maintained’ trajectories to the odds of following the ‘discontinued’ trajectory (as the reference) in individuals with different characteristics. Cox regression models and logistic regression models were run within age categories, and were first adjusted for sex, and then for all covariates simultaneously. Finally, in a sensitivity analysis, we checked the robustness of our individual treatment period definition by reducing the gap between two consecutive dispensations from ‘not exceeding 6 months’ to ‘not exceeding 4.5 months’ (i.e., the grace period, which originally was equal to the supply period, was then reduced by half). We compared the proportions of long-term BZDR users identified by both treatment period definitions. All tests employed two-tailed significance set at p < 0.05. Data management and analyses were performed using SAS, version 9.4 (SAS Institute Inc.) and STATA, version 16.1 (StataCorp LLC, College Station, TX, USA).

## Results

As reported in Supplementary Table S3, in the 0–17 years age group, the majority were dispensed a single BZD-anxiolytic at the treatment initiation (66.8%), with diazepam being most dispensed first drug; while in the groups aged 18–64 and ≥ 65 years, the most common first dispensation was for a single Z-drug (52.6% and 45.0%, respectively), with zopiclone as the top initial drug in both groups. The proportion of individuals with multiple initial BZDRs increased with age from 7.1% in 0–17-year-olds, to 16.4% in those aged 18–64, and to 25.3% in ≥ 65-year-old group.

### Long-term versus short-term BZDR use

Among 0–17-year-olds, the proportion of long-term BZDR use reached 20.7%, raising to 41.0% and 57.4% in 18–64 and ≥ 65-year-olds, respectively (Supplementary Table S3). The sensitivity analysis with more stringent definition of individual treatment period led to similar proportions of long-term BZDR use (18.7%, 38.2%, and 54.2% in 0–17, 18–64, and ≥ 65-year-olds, respectively) (Supplementary Table S4). The proportions of incident BZDR-recipients who progressed to long-term use immediately after treatment initiation also increased with age and corresponded to 16.0%, 33.2%, and 46.1% of all individuals aged 0–17, 18–64, and ≥ 65 years, respectively. Further, individuals with ‘immediate’ long-term BZDR use represented a vast majority of those defined as ever having long-term BZDR use under the study period (77.9%, 80.8%, and 80.4% of long-term users aged 0–17, 18–64, and ≥ 65 years, respectively) (Supplementary Table S3).

Cox proportional hazards regression modelling revealed significant associations of several factors of interest with the risk of long-term BZDR use, and some of these factors were shared across age groups (Supplementary Table S5-S7). However, out of numerous significant associations that were observed in the models minimally-adjusted for sex, only few remained significant in fully-adjusted models. In particular, one of the strongest associations with long-term BZDR use was observed in patients with multiple BZDRs prescribed at treatment initiation, with fully-adjusted hazard ratio (aHR) ranging 3.29–3.63 in different age groups. Other shared predictors included the use of initial single BZD-hypnotic/sedative, Z-drug, or BZD-antiepileptic (aHRs 1.30–2.29 across ages), if compared to patients with a single initial BZD-anxiolytic, as well as being dispensed other medications during 3 months before BZDR initiation (aHRs 1.12–2.70 across ages).

Associations with some factors were specific for certain age groups. Among BZDR-recipients aged 0–17, several individual characteristics were inversely associated with the risk of long-term BZDR use, but the associations were weak and require cautious interpretation (Supplementary Table S5). Among 18–64-year-olds, strong associations with the risk of long-term BZDR use were found in patients with history of substance use disorders (SUD) and somatic multimorbidity, those with the initial prescription from multiple prescribers at different care levels, and individuals with prior disability pension (Supplementary Table S6). For patients aged ≥ 65 years, somatic multimorbidity and initial prescription from multiple prescribers at different care levels were notable associated with long-term BZDR use (Supplementary Table S7). It should be mentioned that in 18–64 and ≥ 65-year-old groups, the initial BZDR prescriptions from multiple prescribers were rarely reported (less than 0.1%).

### Trajectories of BZDR use and characteristics of trajectory-group membership

In individuals with at least 5-year follow-up data (n = 337,799), the proportion of members in the ‘discontinued’ trajectory was the largest among those aged 0–17 years (75.0% of 5065 recipients) and reduced with age to 51.0% of 219,316 recipients in aged 18–64, and to 39.3% of 113,418 recipients aged ≥ 65-years (Fig. 2). In all age groups, the ‘discontinued’ trajectory members used BZDRs for no more than 100–150 days during the first year, for a much shorter period during the next year, and then stopped using BZDRs (thus, the ‘discontinued’ trajectory was similar to the dichotomously measured ‘*short-term BZDR use*’ in the previous section). By contrast, a considerable increase with age was seen in proportions of individuals assigned to the ‘maintained’ trajectory (4.6%, 7.3%, and 36.7% among 0–17, 18–64, and ≥ 65-year-olds, respectively). The corresponding proportions of the ‘decreasing’ and ‘slow decreasing’ trajectory fluctuated across ages. Of note, in all age groups, members of the ‘decreasing’, ‘slow decreasing’, and ‘maintained’ trajectories used BZDR for nearly 300–350 days during one or several years (thus, these 3 trajectories overlapped with the dichotomously measured ‘*long-term BZDR use*’ in the previous section).

**Fig. 3:**
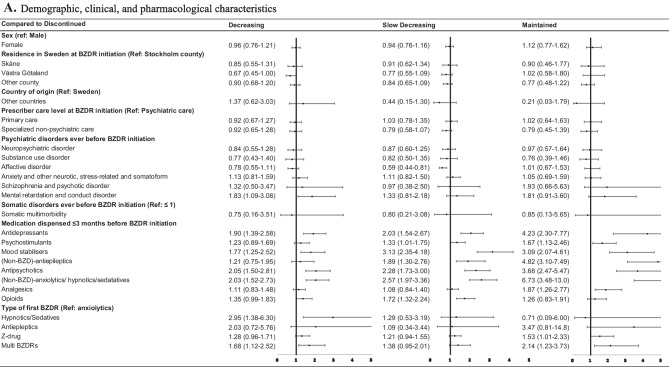

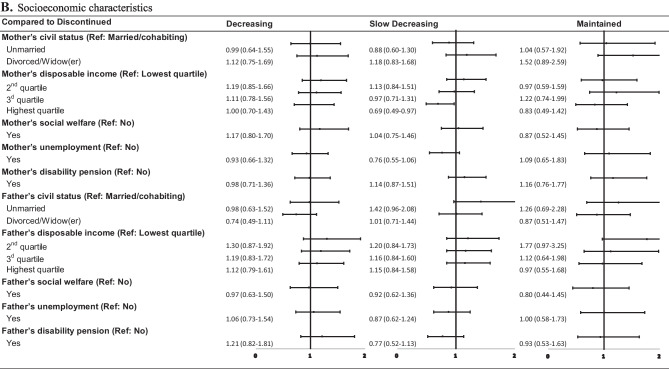
Fully-adjusted odds ratio and 95% confidence intervals for demographic, clinical, and pharmacological characteristics (**A**) and socioeconomic characteristics (**B**) associated with the “decreasing”, “slow decreasing”, and “maintained” trajectories in comparison with the “discontinued” trajectory within the age group 0–17 years at the initiation of BZDR treatment. BZD, Benzodiazepines; BZDR, benzodiazepines and Z-drugs

Multinomial logistic regression revealed factors associated with distinct trajectories. Sex-adjusted modelling results are reported in Supplementary Tables S8-S10 with several individuals and prescribing-related factors showing significant association with the membership in distinct trajectories. In fully-adjusted models, only a few variables remained significant. Thus, Fig. 3 reports the results of fully-adjusted odds ratio (aOR) from multinomial regression for the group aged 0–17. In this age group, the membership in all three trajectories – the ‘decreasing’, ‘slow decreasing’, and ‘maintained’ – was associated with dispensing other medications during 3 months before BZDR initiation (aORs ranging 1.72–6.77 across trajectories with the strongest associations observed for the ‘maintained’ trajectory), if compared to the ‘discontinued’ trajectory membership. Also, the membership in the ‘decreasing’ and ‘maintained’ trajectories was associated with the use of multiple initial BZDRs (aOR = 1.68; 95% CI, 1.12–2.52 and aOR = 2.14; 95% CI, 1.23–3.73, respectively). Among the trajectory-specific factors, the use of the initial single BZD-hypnotic/sedative was associated with the ‘decreasing’ trajectory (aOR = 2.95; 95% CI, 1.68–6.30), while the use of the initial Z-drug was associated with the ‘maintained’ trajectory (aOR = 1.53; 95% CI, 1.01–2.33).

**Fig. 4:**
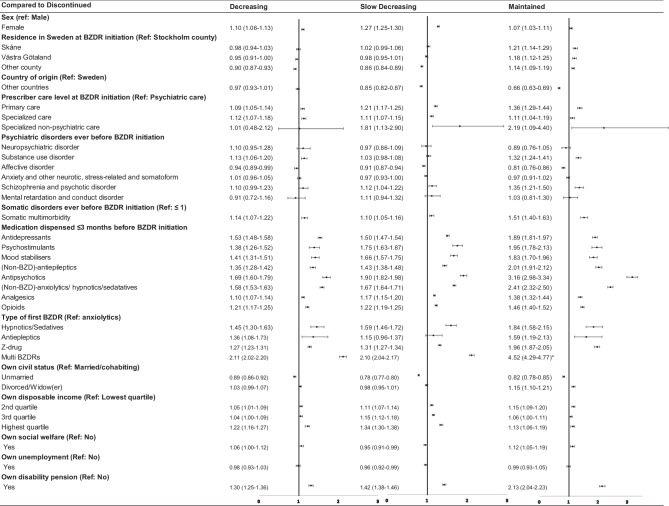
Fully-adjusted odds ratio and 95% confidence intervals for demographic, clinical, pharmacological, and socioeconomic characteristics associated with the “decreasing”, “slow decreasing”, and “maintained” trajectories in comparison with the “discontinued” trajectory in the age group 18–64 years at the initiation of BZDR treatment. *Graphical representation of odds ratio and 95% confidence intervals for multiple initial benzodiazepines and/or z-dugs for the ‘maintained’ trajectory *vs* ‘discontinued’ trajectory is not visible in the figure. BZD, Benzodiazepines; BZDR, benzodiazepines and Z-drugs

As reported in Fig. 4, for the 18–64 years age group, the ‘decreasing’, ‘slow decreasing’, and ‘maintained’ trajectory members also shared some characteristics (with the strongest associations seen for the ‘maintained’ trajectory). These shared characteristics included female sex, primary care or specialized non-psychiatric care as healthcare levels to issue the first BZDR prescription, history of somatic multimorbidity, the use of the initial single BZD-hypnotic/sedative, BZD-antiepileptic, Z-drug, or multiple initial BZDRs, being dispensed other medications within 3 months before BZDR initiation, the second lowest and the highest income quartiles, and disability pension. Prescriptions from multiple prescribers were noted in its association with the increased odds of following ‘slow decreasing’ and ‘maintained’ trajectories. The unmarried civil status seemed to protect from following any of these 3 trajectories. Some factors were specific for the ‘maintained’ trajectory, among which the strongest associations were revealed for a history of SUD, and schizophrenia and psychotic disorders.

**Fig. 5:**
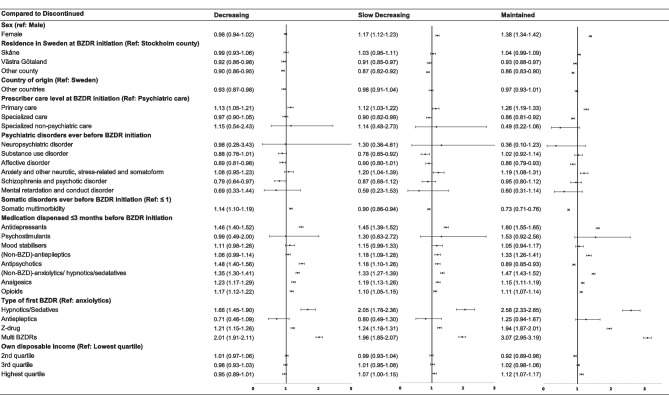
Fully-adjusted odds ratio and 95% confidence intervals for demographic, clinical, pharmacological, and socioeconomic characteristics associated with the “decreasing”, “slow decreasing”, and “maintained” trajectories in comparison with the “discontinued” trajectory in the age group ≥ 65 years at the initiation of BZDR treatment. BZD, Benzodiazepines; BZDR, benzodiazepines and Z-drugs

For the ≥ 65 years age group (Fig. 5), the shared factors that were associated with increased odds of the ‘decreasing, ‘slow decreasing’, and ‘maintained’ trajectories included primary care as the healthcare level to initiate BZDR treatment, the initial BZD-hypnotic/sedative, Z-drug, or multiple initial BZDRs, as well as dispensing other medications during 3 months before BZDR initiation. A history of somatic multimorbidity was associated with the increased odds of following the ‘decreasing’ trajectory, but inversely associated with the ‘slow decreasing’ and ‘maintained’ trajectories. Also, the highest income quartile was exclusively associated with the ‘maintained’ trajectory.

## Discussion

Our population-based registered-based study on BZDR dispensing (as a proxy for BZDR use) among incident recipients resulted in several principal findings. First, across all age groups, we observed considerable proportions of long-term BZDR users (ranging from 20.7% to 57.4% between the youngest and the oldest groups). Also, individuals who progressed to long-term use immediately after treatment initiation represented a substantial part of all BZDR-recipients and accounted for a vast majority of long-term BZDR users within each age group. Studies on incident BZDR-recipients from other countries reported similar but slightly lower figures, although a direct comparison was difficult due to methodological differences, variations in definitions of long-term BZDR use (e.g., varying between 6–12 months of use with the gaps of different lengths), and the exact BZDRs assessed [20–23, 29, 35, 37–39, 51]. For example, a Finnish study reported 34% and 54% of long-term BZDR users among 18–64 and ≥ 65-year-old incident recipients, respectively, and nearly 20% and 30% of the ‘immediate’ long-term users in the same age groups [25]. Studies from the US, Japan, and Germany identified long-term BZDR use in 9%-20% of 18–64-year-old [32, 41], and in 12%-31% of ≥ 65 years old patients [23, 24], with figures reaching up to 80% among the elderly recipients [40–42]. Data on incident BZDR use in youth are scarce, but a study from the US observed long-term BZD use (without Z-drugs) in 10% of children and 12% of adolescents [26], which is lower than our figures for 0–17-year-olds, that again may partly be due to methodological differences between studies.

Second, we identified several factors associated with a risk of developing long-term BZDR use among incident recipients (measured as long-term *vs* short-term use). For all age groups, the most notable associations were found for prescribing-related factors, including multiple BZDRs at the treatment initiation and the single initial BZD-hypnotic/sedative, BZD-antiepileptic, and Z-drug, as well as concurrent dispensing of other medications. The use of multiple initial BZDRs is widely recognised as a strong predictor of long-term BZDR use [25, 32, 40], and strictly discouraged by clinical guidelines, which advocate for monotherapy at the lowest effective dose. When single initial BZDRs were assessed, it appeared that all types of the initial BZDs and Z-drugs, if compared to BZD-anxiolytics (which was substantially represented by diazepam in the youngest group and by oxazepam and diazepam in older ages), were associated with long-term BZDR use. Other studies vary in their reports on what types of single initial BZDR(s) are more predictive of developing long-term BZDR use, e.g., BZD-hypnotics/sedatives [39], BZD-hypnotics/sedatives and Z-drugs [32, 41] (while at least in one study from Japan, the initial single BZD-hypnotics/sedatives and Z-drugs were reported to prevent long-term BZDR use [40]); although the results strongly depend on the drug(s) relative to what the association is assessed. Furthermore, an observed association of long-term BZDR use with concurrent dispensing of BZDR and other psychotropic, antiepileptic and analgesic drugs were in line with other reports [25, 38]. Such co-dispensing might be indicative of patients’ symptom severity; yet, the guidelines strongly indicate the limited duration of BZDR treatment if prescribed concurrently with other medications [10, 52–54], with a particular attention to co-dispensing opioids and BZDRs as two addictive respiratory depressants [55–57]. This emphasizes the need for close monitoring of potential co-prescribing throughout the whole course of BZDR treatment. Also, a simultaneous dispensation of several BZDR prescriptions issued by different prescribers showed strong associations with long-term use in individuals aged 18–64 and ≥ 65 years, although the proportions of patients with overlapping prescriptions were small. Multiple-prescriber BZDR prescriptions may reflect the presence of severe comorbidities (if patients are treated by different healthcare providers) or a ‘doctor-shopping’ behaviour, but both scenarios may result in dependence and overdose [49, 58–60], and require close monitoring by prescribers. In contrast to the prescribing-related factors, individual characteristics were less consistently and to much lower extent associated with long-term BZDR use in all age groups. Our findings of associations of long-term BZDR use with somatic multimorbidity, SUD, disability pension (in adults) as well as no association with gender (in all ages) were in line with some studies on incident BZDR use [22, 35, 36, 61, 62], while there were inconsistencies regarding the role of psychiatric disorders and income (in all ages) [23, 29, 36, 38, 63].

Third, among individuals with 5-year follow-up data, the ‘discontinued’ trajectory members represented the highest proportions of BZDR-recipients in each age group, although such proportions reduced with age (from 75.0% in youths to 39.3% in the elderly). One of the most notable features of the ‘discontinued’ trajectory in all age groups was that the members stopped dispensing BZDRs relatively soon after the treatment initiation and with a duration of use never exceeded 6 months per year. By contrast, for those who used BZDR chronically for several years after the initiation (for approximately 300–350 days per year), it took 3 or 4 years to reduce BZDR use to less than 6 months per year, as it was seen for the ‘decreasing’ and ‘slow decreasing’ trajectories, respectively, or BZDR use never dropped below 6 months per year, as reported for the ‘maintained’ trajectory. The ‘maintained’ trajectory group requires special attention. Already in the youngest group, about 5% of recipients were persistent in BZDR use for approximately 300 days per year for several years with similar figure found for the age group of 18–64 years (over 7%). It is particular worrisome that over 36% of ≥ 65-year-olds were observed to follow the ‘maintained’ trajectory given the reported associations of chronic BZDR use in elderly with a high risk of fall [64], fractures [65], possible risk of dementia [66], as well as the reports on BZDRs as being one of the most commonly inappropriately prescribed medication in this age group [67].

Fourth, we identified several predictors of distinct trajectory groups. In comparison to the ‘discontinued’ trajectory, in all age groups, the prescribing-related factors, including multiple initial BZDRs and co-dispensing of other psychotropics, antiepileptics and analgesics, were most pronouncedly associated with the ‘decreasing’, ‘slow decreasing’, and ‘maintained’ trajectories in fully-adjusted models, and such associations were in most cases stronger for the ‘maintained’ trajectory. Similar findings regarding chronic use trajectories were reported by Verger et al. in their studies on over time use of BZD-anxiolytics [40] and BZD-hypnotics and Z-drugs [41] in individuals aged 50–85. With respect to a single initial BZDR, the associations varied in strength between ages and trajectories, but were more commonly seen for BZD-hypnotics/sedatives and Z-drugs. BZDR prescribing initiated at primary care was associated with the ‘decreasing’, ‘slow decreasing’, and ‘maintained’ trajectories in individuals aged 18–64 and ≥ 65 years (with the strongest associations again seen for the ‘maintained’ trajectory). The role of clinical factors in shaping distinct trajectories was less clear. No consistent associations with a history of psychiatric or somatic disorders were observed in the youngest BZDR-recipients that was different from study by Yeh et al. [27] on adolescents, where chronic BZDR use trajectory was reported to be predicted by a history of psychoses. In our study, prior diagnoses of SUD, schizophrenia and psychotic disorders and records of somatic multimorbidity were notably associated with the ‘maintained’ trajectory group among BZDR-recipients aged 18–64, while in the older individuals, somatic multimorbidity appeared to be inversely associated with the ‘maintained’ trajectory. This echoed the results reported by Verger et al. [40, 41] where trajectories of chronic use of BZD-anxiolytics and BZD-hypnotics and Z-drugs in adults and the elderly were associated with long-standing psychiatric illnesses with less consistency towards somatic illnesses. Finally, socioeconomic factors appeared to be important for shaping ‘decreasing’, ‘slow decreasing’, and ‘maintained’ trajectories in adults aged 18–64, that particularly refers to receiving disability pension prior to BZDR initiation.

Altogether, our findings highlight the importance of prescribing caution, which should be in place already at the initiation of BZDR treatment. Indeed, numerous pharmacoepidemiological studies from different countries, where clinical guidelines on BZDR treatment are similar and agree on advocating the shortest treatment duration for a limited number of indications (e.g., [6–10, 18, 53]), converged to suggest that the high rates of long-term BZDR use may originate from the lack of prescribing caution [20–23, 29, 35, 37–39, 51]. This may reflect a combination of interrelated factors as, for example: i) a diversity in prescribers’ perceptions and knowledge of BZDR risks and benefits, and in their attitudes to BZDR prescribing (e.g., due to a need of maintaining a good doctor-patient relationship and meeting patients’ expectations to receive BZDRs [68–72]); ii) underuse of treatment alternatives (e.g., due to problems with motivating patients to accept non-pharmacological options [71, 73]); iii) prescribing BZDR ‘off-label’ for conditions with weak empirical evidence of risk–benefit ratio [74–77]; and iv) barriers for discontinuation of long-term use (e.g., due to insufficient prescribers’ experience on navigating and monitoring deprescribing process, and concerns on managing withdrawal symptoms [78, 79]). Overall, these concerns emphasize a need for support to prescribers through practice guidelines for decision-making process at each stage of BZDR treatment, including treatment initiation, prevention of transition from new recipients to long-term users, and facilitation of treatment discontinuation. The support for decision -making may in particular be important for prescribers at primary care and non-psychiatric specialty settings. Also, the fact that our findings mainly implicate prescribing-related factors to be associated with ever developing long-term BZDR use and the risk of following the trajectories other than ‘discontinued’ can be useful for clinicians and decision-makers as they indicate the direction for preventive measures aiming at increase in prescribing caution.

### Strengths and limitations

The study strengths include the use of population-based registers that minimizes the risk of sampling error, reporting error and recall bias, and ensures generalizability of the results at the national level. The PDR covers all dispensed drugs regardless of reimbursement status and the service provider characteristics, which makes our data representative of the prescribing practices across all healthcare levels in Sweden. Several limitations should also be acknowledged. First, our analyses rest on dispensation data and, hence, cannot be certain that the medication had been used in proximity to the date of dispensation and by the person it had been prescribed to. Based on prior literature, we assumed that the proportion of individuals who collected, but did not use BZDRs at all or not in proximity to dispensation date, could be minor, if any, and did not vary across the study period [80]. Second, the PDR only covers the period from July 2005, making it impossible to collect information on BZDR prescriptions dispensed prior to that date. With the use of 1.5-year washout period, we attempted to distinguish prevalent and incident user, although this cannot guarantee that the latter group was totally naïve to BZDR use. Third, the PDR does not contain data on the indications for prescriptions and the NPR does not include the diagnosed made at primary care services, which together could have resulted in observing lower proportion of individuals with psychiatric and somatic disorders and, in turn, in less clarity on associations between comorbid diagnoses and BZDR use. Fourth, data on the illegally purchased or recreationally used BZDRs, which are reported for Sweden [81], were not available to us. This could have biased the observed associations if such use was present in certain groups of BZDR-recipients (in addition to the use of the prescribed BZDRs). Lastly, the clinical data retrieved from the NPR were limited to the diagnoses coded with the ICD-10^th^ revision. This may have led to missing psychiatric or somatic disorders if these diagnoses were recorded in the NPR before ICD-10 introduction in 1997 without being mentioned again later.

## Conclusions

A substantial proportion of new BZDR-recipients in Sweden become long-term medication users, particularly in older age, and the majority of long-term BZDR users progress to such pattern of use immediately after treatment initiation. Furthermore, a considerable proportion of individuals maintain long-term BZDR use for several years with slow reduction in the duration of treatment. Prescribing-related factors, rather than individual-user factors, are the main contributors to long-term BZDR use and in maintaining such pattern of use over time. The findings highlight the importance of raising awareness and providing support to prescribers to make evidence-based decisions on initiation and monitoring BZDR treatment across the lifespan.

## Supplementary Information

Below is the link to the electronic supplementary material.

Supplementary file1 (DOCX 160 KB)

## Data Availability

Sharing of the individual-level data is restricted by Swedish data protection laws and data underlying the reported findings cannot be deposited in publicly accessible archives. In this study, data were obtained from the Prescribed Drug Register, the National Patient Register, and the Cause of Death Register held by the Swedish National Board of Health and Welfare (Socialstyrelsen; http://www.socialstyrelsen.se/english), and the Total Population Register, the Multi Generation Register, the Longitudinal Integration Database for Health Insurance and Labour Market Studies, and the Small Areas for Market Statistics maintained by Statistics Sweden (SCB; http://www.scb.se/en/). For further information or enquiries about access to the data, any interested parties can contact the data owners, Socialstyrelsen via registerservice@socialstyrelsen.se and SCB via information@scb.se.
