# Supplementary material for: Factors associated with long-term benzodiazepine and Z-drug use across the lifespan and 5-year temporal trajectories among incident users: a Swedish nationwide register-based study
Source: Eur J Clin Pharmacol. 2023 Jun 9;79(8):1091–105. doi: 10.1007/s00228-023-03515-2 (PMC10361867; doi:10.1007/s00228-023-03515-2)
Supplement: Supplementary file 1 — Supplementary file1 (DOCX 160 KB) [file 228_2023_3515_MOESM1_ESM.docx]

**Supplementary Materials**

**Page**

**Note 1**. The Swedish nationwide registers and databases used in the study ..................................................... 1

**Note 2**. Building trajectories by the group-based trajectory modelling GBTM ................................................ 4

**Table S1**. ATC-codes for BZDR and other medication .................................................................................... 5

**Table S2**. ICD-10 codes and ATC-codes for disorders ..................................................................................... 6

**Table S3.** Distribution of distinct BZDR drugs at initiation, ............................................................................ 7

proportions of long-term BZDR users and ‘immediate’ long-term BZDR users

**Table S4**. Proportions of short-term and long-term BZDR users ..................................................................... 8

defined using the ‘original’ and ‘new’ definitions of individual treatment period

**Table S5**. HR (95% CI) for long- vs short-term BZDR use in association ..................................................... 10

with baseline characteristics in age group of 0-17 years

**Table S6**. HR (95% CI) for long- vs short-term BZDR use in association ..................................................... 13

with baseline characteristics in age group of 18-64 years

**Table S7**. HR (95% CI) for long- vs short-term BZDR use in association ..................................................... 15

with baseline characteristics in age group of ≥65: main and sensitivity analysis

**Table S8**. Trajectory groups of BZDR use and OR (95% CI) ......................................................................... 17

adjusted for sex in individuals aged 0-17 (n=5065).

**Table S9**. Trajectory groups of BZDR use and OR (95% CI) ......................................................................... 19

adjusted for sex in individuals aged 18-64 (n=219,316).

**Table S10**. Trajectory groups of BZDR use and OR (95% CI) ....................................................................... 21

adjusted for sex in individuals aged ≥65 (n=113,418).

**Supplementary Note 1. The Swedish nationwide registers and databases used in the study**

*The Swedish Prescribed Drug Register* encompasses data on prescribed medications dispensed across all pharmacies in Sweden since July 2005 onwards, registered using Anatomical Therapeutic Chemical (ATC) Classification System codes, along with dosage, dispensed amount, dispensation date, and prescriber’s characteristics [1]. The register does not include treatment indication and medications administered in hospitals. From this register, we retrieved data on the initial BZDR, data on other psychotropic, antiepileptic, and analgesic medications, if dispensed within 3 months prior to BZDR initiation, and information on healthcare level, where the first BZDR was prescribed (i.e., primary care, psychiatric care, specialized non-psychiatric care, and multiple prescribers if several prescriptions issued by different healthcare services were simultaneously filled in).

*The National Patient Register* captures diagnostic information from somatic and psychiatric inpatient care (covered since 1969 and 1973, respectively) and specialist outpatient care (since 2001), based on the Swedish version of the International Classification of Diseases, Eighth Revision (ICD-8) (1969-1986), ICD-9 (1987-1996), and ICD-10 (1997-onwards) [2]. The register was validated for an array of diagnoses with an overall positive predictive value of 85-95% and up to 97% for psychiatric disorders [3-6]. Data on diagnosed psychiatric and somatic disorders, if recorded between 1997 (when the International Classification of Diseases, Tenth Edition was introduced) and the first BZDR dispensation, were retrieved from this register. Data from this register were also used to restrict the study population to individuals without the lifetime diagnosis of epilepsy as well as to gain information on individuals who were hospitalized for longer than 90 days during a washout period or ever before the first BZDR dispensation and those with >90-day hospitalization within 6 months after the first dispensation, since long hospitalization data were used to form the exclusion criteria.

*The Cause of Death Register* includes information on all deaths of Swedish residents, occurring in Sweden or abroad, with dates and the international version of the ICD codes for underlying and contributory causes of deaths since 1952 [7]. Death data were collected from this register to be use in the exclusion criterion (if occurred within 6 months after the first dispensation) and as a censoring event in Cox proportional hazards regression models.

*The Total Population Register* records demographic data of all Swedish inhabitants since 1968, and *the Migration Register* captures migration in and out of Sweden [8]. From the Total Population Register, data on individual’s age at the first BZDR dispensation, sex and country of birth were collected, while information on migration was retrieved from the Migration Register, which is part of Total Population Register.

*The Multi-Generation Register* contains information on biological and adopted parents of all individuals who was born in Sweden from 1932 onwards or has ever been registered in the country since 1961 [9]. With the mother as informant, the father is defined as the mother’s husband at the time of birth or the man acknowledged as the father by unmarried mothers. The register spans over five generations and contains data on 100% of mothers and 98% of fathers for those born in Sweden since 1961 [9]. The register was used it to identify and link the biological parents to study participants aged 0-17 years (in order to further collect parental socioeconomic data, since some of such data are not available as ‘own’ characteristics at the age below 18 years).

*The Longitudinal Integration Database for Health Insurance and Labour Market Studies* (LISA, in its Swedish acronym), since 1990 provides annual socioeconomic data for all Swedish residents age 16 years and above [10]. From the LISA register, information on civil status, disposable income, unemployment, disability pension, and social welfare during the year before BZDR initiation (or the nearest year available) was collacted. For individuals with BZDR treatment initiated below age 18, the corresponding socioeconomic data were collected from LISA separately for mothers and fathers (as described above). For those aged ≥65 years, data on unemployment, disability pension, and social welfare were not applicable and, therefore, were not retrieved.

*The Small Areas for Market Statistics (SAMS)*, since 1982 annually records residential information for all Swedish residents by connecting individuals to a specific neighborhood through the addresses registered with the tax authorities at the end of each calendar year [11]. Information on residence in Sweden at the time of BZDR initiation (or the nearest year available) was ascertained from this database.

The Prescribed Drug Register, The National Patient Register, the Cause of Death Register are held by the Swedish National Board of Health and Welfare, and the Total Population Register, the Multi Generation Register, the LISA, and the SAMS are maintained by Statistics Sweden.

**References**

1. Wettermark B, Hammar N, Fored CM, Leimanis A, Otterblad Olausson P, Bergman U, Persson I, Sundstrom A, Westerholm B, Rosen M. The new Swedish Prescribed Drug Register--opportunities for pharmacoepidemiological research and experience from the first six months. *Pharmacoepidemiol Drug Saf* 2007;16(7):726-735.

2. Ludvigsson JF, Andersson E, Ekbom A, Feychting M, Kim JL, Reuterwall C, et al. External review and validation of the Swedish national inpatient register. *BMC Public Health* 2011;11:450.

3. Dalman C, Broms J, Cullberg J, Allebeck P. Young cases of schizophrenia identified in a national inpatient register--are the diagnoses valid? *Soc Psychiatry Psychiatr Epidemiol* 2002;37(11):527-31. doi: 10.1007/s00127-002-0582-3.

4. Ekholm B, Ekholm A, Adolfsson R, Vares M, Osby U, Sedvall GC, et al. Evaluation of diagnostic procedures in Swedish patients with schizophrenia and related psychoses. *Nord J Psychiatry* 2005;59(6):457-464. doi: 10.1080/08039480500360906.

5. Ruck C, Larsson KJ, Lind K, Perez-Vigil A, Isomura K, Sariaslan A, et al. Validity and reliability of chronic tic disorder and obsessive-compulsive disorder diagnoses in the Swedish National Patient Register. *BMJ Open* 2015;5(6):e007520. doi: 10.1136/bmjopen-2014-007520.

6. Sellgren C, Landen M, Lichtenstein P, Hultman CM, Langstrom N. Validity of bipolar disorder hospital discharge diagnoses: file review and multiple register linkage in Sweden. *Acta Psychiatr Scand* 2011;124(6):447-453. doi: 10.1111/j.1600-0447.2011.01747.x.

7. Brooke HL, Talback M, Hornblad J, Johansson LA, Ludvigsson JF, Druid H, et al. The Swedish cause of death register. *Eur J Epidemiol* 2017;32(9):765-773.

8. Ludvigsson JF, Almqvist C, Bonamy AK, Ljung R, Michaelsson K, Neovius M, et al. Registers of the Swedish total population and their use in medical research. Eur J Epidemiol 2016;31(2):125-136.

9. Ekbom A. The Swedish Multi-generation register. *Methods Mol Biol* 2011;675:215-220.

10. Ludvigsson JF, Svedberg P, Olén O, Bruze G, Neovius M. The longitudinal integrated database for health insurance and labour market studies (LISA) and its use in medical research. *Eur J Epidemiol* 2019;34(4): 423-437.

11. Statistics Sweden. Available at [www.scb.se](http://www.scb.se)

**Supplementary Note 2. Building trajectories by the group-based trajectory modelling GBTM**

The GBTM determines the number of trajectories that best fit the data, and assigns individuals into the meaningful groups for which they show statistically similar developmental course. In other words, the groups are not defined *a priory*, but GBTM assigns individuals to the group for which they have the highest probability [1, 2]. In our study, the trajectories were built using the Bayesian information criterion (BIC) for choosing the number of trajectories and their shapes (zero-order, linear, quadratic, cubic or higher). Within each age group, the best fitted models were identified as 4-group trajectories.

The best fitted models for group based trajectory modelling within each age group

|  | **Age group 0-17 years** | **Age group 18-64 years** | **Age group of 65 years and older** |
| --- | --- | --- | --- |
| Number of groups | 4 | 4 | 4 |
| Trajectory shapes | 2 2 1 2 | 3 2 3 3 | 3 3 2 2 |
| BIC for the total number of participants | -46940.46 | -2229073.19 | -1255819.36 |
| BIC for the total number of observations | -46928.39 | -2229057.9 | -1255804.88 |

*Note*: Trajectory shapes; 1 = linear; 2 = quadratic; 3=cubic. BIC = Bayesian information criterion

**References**

1. Nagin D. Analysing developmental trajectories: a semiparametric, group-based approach. *Psychological Methods* 1999;4:139-157.

2. Nagin DS, Odgers CL. Group-based trajectory modeling in clinical research. *Annu Rev Clin Psychol* 2010;6:109-38.

**Supplementary Table S1**. Anatomical Therapeutic Chemical Classification System codes (ATC-codes) retrieved from the Prescribed Drug Register (PDR) for BZDR and other medication.

| **Medication** | **ATC-codes** | **Comments** |
| --- | --- | --- |
| **Benzodiazepines and benzodiazepine-related drugs (BZDR) retrieved from the PDR** | | |
| **Benzodiazepine derivatives in antiepileptics** |  |  |
| Clonazepam | N03AE01 |  |
| **Benzodiazepine derivatives in anxiolytics** |  |  |
| Diazepam | N05BA01 |  |
| Oxazepam | N05BA04 |  |
| Clorazepate | N05BA05 |  |
| Lorazepam | N05BA06 |  |
| Bromazepam | N05BA08 |  |
| Clobazam | N05BA09 |  |
| Alprazolam | N05BA12 |  |
| **Benzodiazepine derivatives in hypnotics/sedatives** |  |  |
| Nitrazepam | N05CD02 |  |
| Flunitrazepam | N05CD03 |  |
| Triazolam | N05CD05 |  |
| Midazolam | N05CD08 |  |
| **Benzodiazepine-related drugs (Z-drugs)** |  |  |
| Zopiclone | N05CF01 |  |
| Zolpidem | N05CF02 |  |
| Zaleplon | N05CF03 |  |
| **Other medication retrieved from the PDR** | | |
| Antidepressants | N06A |  |
| Psychostimulants (centrally acting sympathomimetics) | N06BA |  |
| Mood stabilisers | N03AF01, N03AF02, N03AG01, N03AX09, N03AN01 |  |
| (Non-BZD)-antiepileptics (i.e., excluding benzodiazepine derivatives) | N03 (except N03AE)^a^ and (except N03AF01, N03AF02, N03AG01, N03AX09, N03AN01)^b^ | ^a^ excluded from non-BZD antiepileptics and used to select BZD derivatives in antiepileptics  ^b^ Excluded from non-BZD antiepileptics and used to select mood stabilizers |
| Antipsychotics | N05A |  |
| (Non-BZDR)-anxiolytics, hypnotics, and sedatives (i.e., excluding benzodiazepine derivatives) | N05B (except N05BA)^c^  N05C (except N05CD, N05CF)^d^ | ^c^ Excluded from non-BZDR anxiolytics/ hypnotics/sedatives and used to select benzodiazepine derivatives in anxiolytics  ^d^ Excluded from non-BZDR anxiolytics/ hypnotics/sedatives and used to select benzodiazepine derivatives in hypnotics/sedatives (N05CD) and benzodiazepine-related drugs (N05CF) |
| Analgesics (non-opioids) | N02B, N02C |  |
| Opioids | N02A |  |

**Supplementary Table S2**. List of Swedish International Classification of Diseases, Tenth Edition codes (ICD-10 codes) in the National Patient Register and Anatomical Therapeutic Chemical Classification System codes (ATC-codes) in the Prescribed Drug register to collect records of psychiatric and somatic disorders.

| **Diagnoses** | **ICD-10 codes** | **Minimal age limit** |
| --- | --- | --- |
| **Psychiatric disorder groups and psychiatric disorders** |  |  |
| *Neuropsychiatric disorders:* |  |  |
| Attention-deficit/hypersensitivity disorder | F90 or *ATC-codes*: N06BA01, N06BA02, N06BA04, N06BA09, N06BA12 | ≥ 3 years |
| Autism spectrum disorders | F84.0, F84.1, F84.3, F84.5, F84.8, F84.9 | ≥ 1 year |
| *Substance use disorders* | F10-F16 and F18-F19 (all except F1x.5) or *ATC-codes*: N07BB, N07BC01, N07BC02, N07BC51 (if N07BC prescribed at psychiatry in- or outpatient care) | ≥ 10 years |
| *Affective disorders:* |  |  |
| Depressive disorders | F32, F33, F34 (except F34.0), F38, F39 | ≥ 6 years |
| Bipolar disorders | F25.0, F30, F31, F34.0 | ≥ 10 years |
| *Anxiety and other neurotic, stress-related and somatoform disorders:* |  |  |
| Anxiety disorders | F40, F41 | ≥ 6 years |
| Obsessive-compulsive disorder | F42 | ≥ 6 years |
| Reaction to severe stress and adjustment disorders | F43 | ≥ 6 years |
| Dissociative, somatoform and other neurotic disorders | F44, F45, F48 | ≥ 6 years |
| *Schizophrenia, schizotypal, and delusional disorders and psychotic disorders* | F20, F21, F22, F23, F24, F25 (except F25.0), F28, F29, F10.5-F16.5 and F18.5-F19.5 | ≥ 10 years |
| *Mental retardation and conduct disorders:* |  |  |
| Mental retardation | F70-F79 | ≥ 1 year |
| Disruptive behaviour disorders | F91 | ≥ 3 years |
| **Somatic disorders^a^** |  |  |
| Epilepsy, status epilepticus^b^ | G40, G41 | No min age limit |
| Nonorganic sleep disorders & insomnias | F51.0, G47.0 | No min age limit |
| Hypertension | I10- I16 | No min age limit |
| Ischemic heart diseases | I20-I25 | No min age limit |
| Coronary heart failure | I42, I43, I50 | No min age limit |
| Stroke and other cerebrovascular disease | I60-I69 | No min age limit |
| Diabetes mellitus | E10-E14 | No min age limit |
| Asthma or chronic obstructive pulmonary disease | J44-J46 | No min age limit |
| Other chronic pulmonary diseases | J40-43, J47 | No min age limit |
| Connective tissue diseases | M05, M06, M32-M34, M35.1, M35.3 | No min age limit |
| All-cause hyperthyroidism | E05.0, E05.1, E05.2, E05.9 | No min age limit |
| Hypothyroidism | E03 | No min age limit |
| Inflammatory bowel disease (Crohn’s disease and ulcerative colitis) | K50, K51 | No min age limit |
| Parkinson’s disorder | G20 | No min age limit |
| All-cause dementia, including Alzheimer’s disease | G30, F00-F03 or *ATC-code*: N06D | No min age limit |
| Multiple sclerosis | G35 | No min age limit |

^a^ All somatic disorders (apart from epilepsy and status epilepticus) are used to analyse multimorbidity

^b^ Used to restrict the study cohort for individuals free from a history of epilepsy

**Supplementary Table S3**. Distribution of distinct BZDR drugs at initiation (with a separate category for multiple BZDRs), proportion of long-term BZDR recipients, and proportion of individuals with long-term BZDR dispensation immediately from initiation within age categories

|  | **Age 0-17 years at BZDR initiation**  **(n=18,484)** | | | | **Age 18-64 years at BZDR initiation**  **(n=590,720)** | | | | **Age 65 years and older at BZDR initiation**  **(n=321,261)** | | | |
| --- | --- | --- | --- | --- | --- | --- | --- | --- | --- | --- | --- | --- |
|  | **All** | **Females** | **Ever long-term use** | **Long-term use from initiation** | **All** | **Females** | **Ever long-term use** | **Long-term use from initiation** | **All** | **Females** | **Ever long-term use** | **Long-term use from initiation** |
|  | **n (%)^a^** | **n (%)^b^** | **n (%)^b^** | **n (%)^b^ / [%]^c^** | **n (%)^a^** | **n (%)^b^** | **n (%)^b^** | **n (%)^b^ / [%]^c^** | **n (%)^a^** | **n (%)^b^** | **n (%)^b^** | **n (%)^b^ / [%]^c^** |
| **Total** | 18,484 (100) | 9458 (51.2) | 3826 (20.7) | 2982 (16.1) [77.9] | 590,720 (100) | 348,049 (58.9) | 242,479 (41.0) | 195,866 (33.2) [80.8] | 321,261 (100) | 189,413 (58.9) | 184,367 (57.4) | 148,276 (46.1) [80.4] |
| **BZD-anxiolytics** | 12,351 (66.8) | 5853 (47.4) | 1340 (10.8) | 848 (6.86) [63.3] | 173,899 (29.4) | 104,697 (60.2) | 47,766 (27.5) | 29,975 (17.2) [62.7] | 90,075 (28.0) | 55,771 (61.9) | 37,400 (41.5) | 23,648 (26.2) [63.2] |
| Diazepam | 11,029 (59.7) | 5030 (45.6) | 1013 (9.2) | 613 (5.5) [60.5] | 55,186 (9.3) | 30.912 (56.0) | 12,668 (22.9) | 6293 (11.4) [49.7] | 17,850 (5.5) | 10,137 (56.8) | 5495 (30.7) | 2456 (13.7) [44.7] |
| Oxazepam | 1093 (5.9) | 671 (61.4) | 247 (22.6) | 163 (14.9) [65.9] | 105,155 (17.8) | 65,913 (62.7) | 30,089 (28.9) | 19,673 (18.7) [65.4] | 69,415 (21.6) | 43,986 (63.3) | 30,556 (44.0) | 20,097 (28.9) [65.8] |
| Clorazepate | 0 | 0 (0) | 0 (0) | 0 (0)[0] | 0 (0) | 0 (0) | 0 (0) | 0 (0) [0] | 0 (0) | 0 | 0 | 0 |
| Lorazepam | 46 (0.2) | 27 (58.7) | 13 (28.3) | 13 (28.2) [100] | 1409 (0.2) | 837 (59.4) | 461 (32.7) | 379 (26.9) [82.2] | 497 (0.1) | 270 (54.3) | 242 (48.7) | 183 (36.8) [82.4] |
| Bromazepam | 0 | 0 | 0 (0) | 0 (0) [0] | 0 (0) | 0 (0) | 0 (0) | 0 (0) [0] | 0 | 0 | 0 | 0 |
| Clobazam | ≤10 | ≤10 | ≤10 | ≤10 | ≤10 | ≤10 | ≤10 | ≤10 | 0 | 0 | 0 | 0 |
| Alprazolam | 178 (0.9) | 124 (66.7) | 65 (36.5) | 57 (32.0) [87.7] | 12,147 (2.1) | 7034 (57.9) | 4548 (37.4) | 3630 (29.9) [79.8] | 2313 (0.7) | 1378 (59.6) | 1107 (47.8) | 912 (39.4) [82.4] |
| **BZD- hypnotics / sedatives** | 195 (1.05) | 104 (53.3) | 52 (26.7) | 38 (19.5) [73.1] | 7234 (1.2) | 3806 (52.6) | 2959 (40.9) | 2267 (31.3) [76.6] | 4580 (1.4) | 2493 (54.4) | 2479 (54.1) | 1690 (36.9) [68.2] |
| Nitrazepam | 82 (0.4) | 44 (53.6) | 34 (41.5) | 23 (28.0) [67.6] | 3160 (0.5) | 1635 (51.7) | 1293 (40.9) | 1005 (31.8) [77.7] | 1552 (0.5) | 848 (54.6) | 812 (52.3) | 589 (37.9) [72.5] |
| Flunitrazepam | 20 (0.1) | ≤10 | ≤10 | ≤10 | 3308 (0.6) | 1758 (53.1) | 1402 (42.4) | 1078 (32.6) [76.9] | 2641 (0.8) | 1433 (54.3) | 1518 (57.5) | 997 (37.7) [65.7] |
| Triazolam | 21 (0.1) | 14 (66.7) | ≤10 | ≤10 | 709 (0.1) | 381 (53.7) | 247 (34.8) | 168 (23.7) [68.0] | 301 (0.1) | 157 (52.2) | 133 (44.2) | 88 (29.2) [66.2] |
| Midazolam | 72 (0.4) | 39 (54.2) | ≤10 | ≤10 | 57 (0.01) | 32 (56.1) | 17 (29.8) | 16 (28.1) [94.1] | 86 (0.03) | 55 (63.9) | 16 (18.6) | 16 (18.6) [100] |
| **BZD-antiepileptics** | 161 (0.9) | 70 (43.5) | 77 (47.8) | 46 (28.6) [59.7] | 1824 (0.3) | 934 (51.2) | 714 (39.1) | 439 (24.1) [61.5] | 815 (0.2) | 387 (47.5) | 310 (38.0) | 182 (22.3) [58.7] |
| Clonazepam | 161 (0.9) | 70 (43.5) | 77 (47.8) | 46 (28.6) [59.7] | 1824 (0.3) | 934 (51.2) | 714 (39.1) | 439 (24.1) [61.5] | 815 (0.2) | 387 (47.5) | 310 (38.0) | 182 (22.3) [58.7] |
| **Z-drugs** | 4462 (24.1) | 2662 (59.7) | 1597 (57.8) | 1395 (31.3) [87.3] | 310,945 (52.6) | 182,746 (58.8) | 124,914 (68.3) | 104,397 (33.6) [83.6] | 144,629 (45.0) | 82,477 (57.0) | 77,994 (53.9) | 63,764 (44.1) [81.7] |
| Zopiclone | 2727 (14.7) | 1674 (61.4) | 940 (34.5) | 790 (28.9) [84.0] | 174,134 (29.5) | 101,545 (58.3) | 67,813 (38.9) | 54,474 (31.2) [80.3] | 91,566 (28.5) | 51,302 (56.0) | 48,535 (53.0) | 38,686 (42.2) [79.7] |
| Zolpidem | 1664 (9.0) | 948 (56.9) | 627 (37.7) | 575 (34.5) [91.7] | 132,821 (22.5) | 78,927 (59.4) | 55,209 (41.5) | 48,067 (36.2) [87.1] | 52,391 (16.3) | 30,779 (58.7) | 29,078 (55.5) | 24,704 (47.1) [84.9] |
| Zaleplon | 71 (0.4) | 40 (56.3) | 30 (42.2) | 30 (42.2) [100] | 3990 (0.7) | 2274 (56.9) | 1892 (47.4) | 1856 (46.5) [98.1] | 672 (0.2) | 396 (58.9) | 381 (56.7) | 374 (55.6) [98.2] |
| **Multiple BZDs and/or Z-drugs^d^** | 1315 (7.1) | 769 (58.5) | 760 (57.8) | 655 (49.8) [86.1] | 96,818 (16.4) | 55,866 (57.7) | 66,126 (68.3) | 58,788 (60.7) [88.9] | 81,162 (25.2) | 48,285 (59.5) | 66,184 (81.5) | 58,992 (72.7) [89.1] |

*Note*: If a certain drug is reported only in 10 or less individuals, the actual frequencies are replaced with ‘≤10’ to mitigate any possibility for backward identification.

^a^ Proportions are estimated within a column, i.e., from the total number of individuals within each age category.

^b^ Proportions are estimated within rows, i.e., out of all individuals with the same BZDR within each age category.

^c^ Proportions of BZDR-recipients with long-term dispensation immediately from initiation are estimated out of the number of all long-term recipients of the same BZDR drug, within each age category.

^d^ Refers to individuals if their first dispensed prescription contained more than one BZDR drug. All abovementioned BZDRs (apart from bromazepam and clorazepate) were present in the ‘multiple BZDRs’ category in different combinations.

*Abbreviation*: BZD, benzodiazepines

**Supplementary Table S4**. Proportions of short-term and long-term BZDR users defined using the ‘original’ and ‘new’ definitions of individual treatment period

|  | **Age group 0-17 years**  **(n=18,484)** | | **Age group 18-64 years**  **(n=590,720)** | | **Age group of 65 years and older**  **(n=321,261)** | |
| --- | --- | --- | --- | --- | --- | --- |
|  | **Short-term BZDR users** | **Long-term BZDR users** | **Short-term BZDR users** | **Long-term BZDR users** | **Short-term BZDR users** | **Long-term BZDR users** |
| ‘Original’ definition for individual treatment period^a^ | 14,658 (79.30) | 3826 (20.70) | 348,241 (58.95) | 242,479 (41.05) | 136,894 (42.61) | 184,367 (57.39) |
| ‘New’ definition for individual treatment period^a^ | 15,031 (81.32) | 3453 (18.68) | 364,994 (61.79) | 225,726 (38.21) | 147,123 (45.80) | 174,138 (54.20) |
| Difference in proportions of long-term BZDR users between ‘old’ and ‘new’ definitions (‘*original’ minus ‘new’*) |  | 2.0% |  | 2.8% |  | 3,2% |

^a^ The ‘original’ definition of individual treatment period = a sequence of BZDR dispensations if the gap between dates of two consecutive dispensations did not exceed **6 months**. With a gap extending beyond 6 months, the next dispensation was considered as the initiation of a new treatment regimen.

^b^ The ‘new’ definition of individual treatment period = a sequence of BZDR dispensations if the gap between dates of two consecutive dispensations did not exceed **4.5 months**. With a gap extending beyond 4.5 months, the next dispensation was considered as the initiation of a new treatment regimen.

Abbreviation: BZDR; benzodiazepines and benzodiazepine-related drugs

**Supplementary Table S5**. Hazard ratios (HR) and 95% confidence intervals (95% CI) for long-term BZDR use versus short-term use in associations with demographic, clinical, pharmacological, and socio-economic characteristics in age group of 0-17 at BZDR initiation

|  | **Short-term users**  **n=14,687** | **Long-term users**  **n=3797** | **Minimally-adjusted for sex** | **Fully-adjusted for all covariates** |
| --- | --- | --- | --- | --- |
|  | **n (%)** | **n (%)** | **HR (95% CI)** | **HR (95% CI)** |
| **Sex** |  |  |  |  |
| Male | 7449 (50.7) | 1577 (41.5) | 1.00 | 1.00 |
| Female | 7238 (49.3) | 2220 (58.5) | **1.38 (1.29-1.48)** | 1.02 (0.95-1.09) |
| **Place of birth** |  |  |  |  |
| Sweden | 14407 (98.1) | 3687 (97.1) | 1.00 | 1.00 |
| Other countries | 280 (1.9) | 110 (2.9) | 1.18 (0.87-1.62) | 0.85 (0.62-1.17) |
| **Residence in Sweden at BZDR initiation^a^** |  |  |  |  |
| Stockholm county | 3348 (22.8) | 895 (23.6) | 1.00 | 1.00 |
| Skåne | 1579 (10.7) | 360 (9.5) | ***0.85 (0.75-0.97)*** | ***0.80 (0.70-0.91)*** |
| Västra Götaland | 2163 (14.7) | 628 (16.5) | 1.06 (0.95-1.18) | ***0.86 (0.77-0.96)*** |
| Other county | 7464 (50.8) | 1871 (49.3) | 0.93 (0.85-1.01) | ***0.83 (0.76-0.90)*** |
| Missing / unclear | 133 (0.9) | 43 (1.1) |  |  |
| **Psychiatric disorders ever before BZDR initiation** |  |  |  |  |
| Neuropsychiatric disorders | 1064 (7.2) | 621 (16.4) | **2.42 (2.21-2.65)** | 1.02 (0.91-1.13) |
| Substance use disorders | 248 (1.7) | 222 (5.9) | **2.81 (2.43-3.24)** | 0.98 (0.85-1.14) |
| Affective disorders | 1037 (7.1) | 809 (21.3) | **2.89 (2.66-3.14)** | ***0.88 (0.80-0.97)*** |
| Anxiety and other neurotic, stress-related and somatoform disorders | 1082 (7.4) | 780 (20.5) | **2.66 (2.44-2.89)** | 1.01 (0.92-1.11) |
| Schizophrenia and psychotic disorders | 70 (0.5) | 72 (1.9) | **3.24 (2.52-4.16)** | 1.12 (0.86-1.45) |
| Mental retardation and conduct disorders | 515 (3.5) | 294 (7.7) | **2.14 (1.89-2.42)** | 1.10 (0.96-1.27) |
| **Somatic disorders ever before BZDR initiation** |  |  |  |  |
| None or 1 somatic disorder | 14572 (99.2) | 3741 (98.5) | 1.00 | 1.00 |
| Somatic multimorbidity (2 or more disorders) | 115 (0.8) | 56 (1.5) | **1.90 (1.46-2.48)** | 0.99 (0.75-1.29) |
| **Type of BZDR at initiation** |  |  |  |  |
| Anxiolytic | 11031 (75.1) | 1320 (34.8) | 1.00 | 1.00 |
| Hypnotic/sedative | 143 (1.0) | 52 (1.4) | **2.68 (2.00-3.60)** | **1.98 (1.47-2.66)** |
| Antiepileptic | 84 (0.6) | 77 (2.0) | **5.42 (4.29-6.84)** | **2.29 (1.80-2.90)** |
| Z-drug | 2871 (19.5) | 1591 (41.9) | **3.97 (3.68-4.29)** | **2.08 (1.90-2.29)** |
| Multiple BZDRs at initiation | 558 (3.8) | 757 (19.9) | **7.73 (7.04-8.49)** | **3.63 (3.28-4.01)** |
| **Medication dispensed ≤3 months before BZDR initiation** |  |  |  |  |
| Antidepressants | 2794 (19.0) | 2248 (59.2) | **4.72 (4.41-5.05)** | **1.83 (1.66-2.02)** |
| Psychostimulants | 1233 (8.4) | 931 (24.5) | **2.99 (2.77-3.23)** | **1.12 (1.02-1.23)** |
| Mood stabilisers | 2116 (14.4) | 1528 (40.2) | **3.21 (3.00-3.43)** | **2.37 (2.19-2.56)** |
| (Non-BZD)-antiepileptics | 684 (4.7) | 979 (25.8) | **4.65 (4.31-5.01)** | **2.70 (2.48-2.94)** |
| Antipsychotics | 880 (6.0) | 1227 (32.3) | **4.92 (4.58-5.29)** | **1.35 (1.23-1.47)** |
| (Non-BZD)-anxiolytics/hypnotics/sedatives | 3107 (21.2) | 2453 (64.6) | **5.24 (4.89-5.62)** | **1.90 (1.73-2.09)** |
| Analgesics | 1782 (12.1) | 986 (26.0) | **2.07 (1.92-2.24)** | **1.12 (1.03-1.23)** |
| Opioids | 1202 (8.2) | 862 (22.7) | **2.55 (2.36-2.77)** | **1.15 (1.04-1.26)** |
| **Prescriber care level at BZDR initiation** |  |  |  |  |
| Psychiatry care | 2927 (19.9) | 1818 (47.9) | 1.00 | 1.00 |
| Primary care | 2870 (19.5) | 728 (19.2) | ***0.48 (0.44-0.53)*** | 0.93 (0.85-1.03) |
| Specialized non-psychiatric care | 8888 (60.5) | 1251 (32.9) | ***0.28 (0.26-0.30)*** | ***0.86 (0.77-0.96)*** |
| Multiple prescribers | ≤10 | 0 | NA | NA |
| **Mother’s social status the year prior to child’s BZDR initiation** |  |  |  |  |
| Married/cohabiting | 7214 (49.1) | 1764 (46.5) | 1.00 | 1.00 |
| Unmarried | 5281 (36.0) | 1088 (28.6) | ***0.86 (0.80-0.93)*** | 1.01 (0.91-1.11) |
| Divorced/widow(er) | 1857 (12.6) | 789 (20.8) | **1.59 (1.46-1.74)** | 1.01 (0.91-1.12) |
| Missing / unclear | 335 (2.3) | 156 (4.1) |  |  |
| **Mother’s disposable income the year prior to child’s BZDR initiation** |  |  |  |  |
| Lowest quartile | 6100 (41.5) | 1182 (31.1) | 1.00 | 1.00 |
| 2^nd^ quartile | 3388 (23.1) | 978 (25.8) | **1.43 (1.31-1.56)** | 0.98 (0.88-1.09) |
| 3^d^ quartile | 2868 (19.5) | 856 (22.5) | **1.47 (1.34-1.61)** | 1.02 (0.91-1.15) |
| Highest quartile | 2039 (13.9) | 646 (17.0) | **1.50 (1.36-1.66)** | 0.99 (0.88-1.11) |
| Missing / unclear | 292 (2.0) | 135 (3.6) |  |  |
| **Mother’s social welfare the year prior to child’s BZDR initiation** |  |  |  |  |
| No | 13075 (89.0) | 3179 (83.7) | 1.00 | 1.00 |
| Yes | 1344 (9.2) | 494 (13.0) | **1.42 (1.29-1.56)** | 1.05 (0.95-1.16) |
| Missing / unclear | 268 (1.8) | 124 (3.3) |  |  |
| **Mother’s unemployment the year prior to child’s BZDR initiation** |  |  |  |  |
| No | 13118 (89.3) | 3319 (87.4) | 1.00 | 1.00 |
| Yes | 1301 (8.9) | 354 (9.3) | 1.03 (0.92-1.15) | 0.98 (0.90-1.06) |
| Missing / unclear | 268 (1.8) | 124 (3.3) |  |  |
| **Mother’s disability pension the year prior to child’s BZDR initiation** |  |  |  |  |
| No | 13381 (91.1) | 3114 (82.0) | 1.00 | 1.00 |
| Yes | 1038 (7.1) | 559 (14.7) | **1.97 (1.80-2.16)** | 1.00 (0.91-1.09) |
| Missing / unclear | 268 (1.8) | 124 (3.3) |  |  |
| **Father’s social status the year prior to child’s BZDR initiation** |  |  |  |  |
| Married/cohabiting | 7166 (48.8) | 1778 (46.8) | 1.00 | 1.00 |
| Unmarried | 5169 (35.2) | 1036 (27.3) | ***0.83 (0.77-0.90)*** | 1.05 (0.93-1.19) |
| Divorced/widow(er) | 1767 (12.0) | 749 (19.7) | **1.57 (1.44-1.71)** | 1.01 (0.91-1.13) |
| Missing / unclear | 585 (4.0) | 234 (6.2) |  |  |
| **Father’s disposable income the year prior to child’s BZDR initiation** |  |  |  |  |
| Lowest quartile | 2774 (18.9) | 548 (14.4) | 1.00 | 1.00 |
| 2^nd^ quartile | 2385 (16.2) | 603 (15.9) | **1.22 (1.09-1.38)** | 1.07 (0.96-1.19) |
| 3^d^ quartile | 4204 (28.6) | 1009 (26.6) | **1.17 (1.06-1.30)** | 0.92 (0.80-1.05) |
| Highest quartile | 4709 (32.1) | 1400 (36.9) | **1.44 (1.30-1.59)** | 1.02 (0.90-1.15) |
| Missing / unclear | 615 (4.2) | 237 (6.2) |  |  |
| **Father’s social welfare the year prior to child’s BZDR initiation** |  |  |  |  |
| No | 13134 (89.4) | 3243 (85.4) | 1.00 | 1.00 |
| Yes | 989 (6.7) | 333 (8.8) | **1.29 (1.15-1.45)** | 0.94 (0.83-1.07) |
| Missing / unclear | 564 (3.8) | 221 (5.8) |  |  |
| **Father’s unemployment the year prior to child’s BZDR initiation** |  |  |  |  |
| No | 13106 (89.2) | 3279 (85.4) | 1.00 | 1.00 |
| Yes | 1017 (6.9) | 297 (7.8) | 1.13 (1.00-1.28) | 1.00 (0.99-1.02) |
| Missing / unclear | 564 (3.8) | 221 (5.8) |  |  |
| **Father’s disability pension the year prior to child’s BZDR initiation** |  |  |  |  |
| No | 13471 (91.7) | 3278 (86.3) | 1.00 | 1.00 |
| Yes | 652 (4.4) | 298 (7.9) | **1.71 (1.52-1.93)** | ***0.91 (0.89-0.94)*** |
| Missing / unclear | 564 (3.8) | 257 (5.8) |  |  |

*Note*: Significant hazard rations are written in bold. Inverse associations are written in Italics. The analyses are conducted among the complete analytical sample, i.e., among the individuals with all data on covariates available. If a certain characteristic is reported only in 10 or less individuals, the actual frequencies are replaced with ‘≤10’ to mitigate any possibility for backward identification.

^a^ Stockholm county – the largest county in Sweden, Skåne – the second largest county in the country, Västra Götaland – the third largest county in Sweden

**Supplementary Table S6**. Hazard ratios (HR) and 95% confidence intervals (95% CI) for long-term BZDR use versus short-term use in associations with demographic, clinical, pharmacological, and socio-economic characteristics in age group of 18-64 at BZDR initiation

|  | **Short-term users**  **n=349,620** | **Long-term users**  **n=241,100** | **Minimally-adjusted for sex** | **Fully-adjusted for all covariates** |
| --- | --- | --- | --- | --- |
|  | **n (%)** | **n (%)** | **HR (95% CI)** | **HR (95% CI)** |
| **Sex** |  |  |  |  |
| Male | 144350 (41.3) | 98321 (40.8) | 1.00 | 1.00 |
| Female | 205270 (58.7) | 142779 (59.2) | 1.01 (1.00-1.02) | 1.01 (1.00-1.01) |
| **Place of birth** |  |  |  |  |
| Sweden | 290879 (83.2) | 200685 (83.2) | 1.00 | 1.00 |
| Other countries | 58741 (16.8) | 40415 (16.8) | 0.99 (0.98-1.01) | ***0.90 (0.89-0.91)*** |
| **Residence in Sweden at BZDR initiation^a^** |  |  |  |  |
| Stockholm county | 87736 (25.1) | 59439 (24.7) | 1.00 | 1.00 |
| Skåne | 46347 (13.3) | 32865 (13.6) | **1.04 (1.02-1.05)** | **1.04 (1.03-1.06)** |
| Västra Götaland | 61975 (17.7) | 44057 (18.3) | **1.03 (1.02-1.05)** | 1.00 (0.99-1.01) |
| Other county | 152837 (43.7) | 104486 (43.3) | 1.00 (0.99-1.01) | ***0.97 (0.96-0.98)*** |
| Missing / unclear | 725 (0.2) | 253 (0.1) |  |  |
| **Psychiatric disorders ever before BZDR initiation** |  |  |  |  |
| Neuropsychiatric disorders | 4996 (1.4) | 6328 (2.6) | **1.65 (1.61-1.70)** | **1.04 (1.01-1.07)** |
| Substance use disorders | 17464 (5.0) | 22233 (9.2) | **1.67 (1.64-1.69)** | **1.14 (1.12-1.15)** |
| Affective disorders | 29950 (8.6) | 32458 (13.5) | **1.48 (1.46-1.50)** | ***0.92 (0.91-0.93)*** |
| Anxiety and other neurotic, stress-related and somatoform disorders | 38751 (11.1) | 36116 (15.0) | **1.33 (1.31-1.34)** | ***0.96 (0.95-0.97)*** |
| Schizophrenia and psychotic disorders | 4601 (1.3) | 6969 (2.9) | **1.77 (1.73-1.82)** | **1.06 (1.03-1.09)** |
| Mental retardation and conduct disorders | 1484 (0.4) | 1633 (0.7) | **1.46 (1.39-1.53)** | 0.95 (0.91-1.00) |
| **Somatic disorders ever before BZDR initiation** |  |  |  |  |
| None or 1 somatic disorder | 334275 (95.6) | 226066 (93.8) | 1.00 | 1.00 |
| Somatic multimorbidity (2 or more disorders) | 15345 (4.4) | 15034 (6.2) | **1.33 (1.31-1.36)** | **1.16 (1.14-1.18)** |
| **Type of BZDR at initiation** |  |  |  |  |
| Anxiolytic | 126675 (36.2) | 47224 (19.6) | 1.00 | 1.00 |
| Hypnotic/sedative | 4297 (1.2) | 2937 (1.2) | **1.63 (1.57-1.69)** | **1.67 (1.61-1.73)** |
| Antiepileptic | 1116 (0.3) | 708 (0.3) | **1.52 (1.41-1.64)** | **1.30 (1.20-1.40)** |
| Z-drug | 186654 (53.4) | 124291 (51.5) | **1.67 (1.66-1.69)** | **1.79 (1.77-1.81)** |
| Multiple BZDRs at initiation | 30878 (8.8) | 65940 (27.4) | **3.70 (3.65-3.74)** | **3.36 (3.32-3.40)** |
| **Medication dispensed ≤3 months before BZDR initiation** |  |  |  |  |
| Antidepressants | 147198 (42.1) | 155510 (64.5) | **2.02 (2.01-2.04)** | **1.63 (1.61-1.64)** |
| Psychostimulants | 5891 (1.7) | 12120 (5.0) | **2.13 (2.09-2.17)** | **1.24 (1.22-1.27)** |
| Mood stabilisers | 8066 (2.3) | 18172 (7.5) | **2.24 (2.21-2.27)** | **1.26 (1.24-1.28)** |
| (Non-BZDR)-antiepileptics | 15494 (4.4) | 30427 (12.6) | **2.12 (2.09-2.15)** | **1.30 (1.29-1.32)** |
| Antipsychotics | 15380 (4.4) | 36955 (15.3) | **2.48 (2.46-2.51)** | **1.58 (1.56-1.61)** |
| (Non-BZDR)-anxiolytics/hypnotics/sedatives | 102356 (29.3) | 127718 (53.0) | **2.10 (2.09-2.12)** | **1.59 (1.57-1.60)** |
| Analgesics | 124242 (35.5) | 116110 (48.2) | **1.44 (1.43-1.45)** | **1.15 (1.14-1.16)** |
| Opioids | 118077 (33.8) | 113704 (47.2) | **1.47 (1.46-1.49)** | **1.25 (1.24-1.26)** |
| **Prescriber care level at BZDR initiation** |  |  |  |  |
| Psychiatry care | 52338 (15.0) | 51470 (21.4) | 1.00 | 1.00 |
| Primary care | 236684 (67.7) | 150890 (62.6) | ***0.72 (0.71-0.73)*** | **1.07 (1.05-1.08)** |
| Specialized non-psychiatric care | 60465 (17.3) | 38535 (16.0) | ***0.72 (0.71-0.73)*** | **1.10 (1.08-1.12)** |
| Multiple prescribers | 116 (0.03) | 178 (0.07) | **1.41 (1.22-1.64)** | **1.32 (1.14-1.54)** |
| Missing / unclear | 17 (0.005) | 27 (0.01) |  |  |
| **Civil status the year prior to BZDR initiation** |  |  |  |  |
| Married/cohabiting | 147468 (42.2) | 96319 (39.9) | 1.00 | 1.00 |
| Unmarried | 149330 (42.7) | 101202 (42.0) | **1.05 (1.04-1.06)** | ***0.93 (0.93-0.94)*** |
| Divorced/widow(er) | 48239 (13.8) | 40252 (16.7) | **1.20 (1.19-1.22)** | **1.05 (1.04-1.06)** |
| Missing / unclear | 4583 (1.3) | 3327 (1.4) |  |  |
| **Disposable income the year prior to BZDR initiation** |  |  |  |  |
| Lowest quartile | 81060 (23.2) | 63083 (26.2) | 1.00 | 1.00 |
| 2^nd^ quartile | 71786 (20.5) | 55283 (22.9) | 0.99 (0.98-1.00) | **1.02 (1.01-1.03)** |
| 3^d^ quartile | 89650 (25.6) | 60087 (24.9) | ***0.89 (0.88-0.91)*** | 1.02 (1.00-1.03) |
| Highest quartile | 105236 (30.1) | 61701 (25.6) | ***0.81 (0.80-0.82)*** | **1.03 (1.02-1.04)** |
| Missing / unclear | 1888 (0.5) | 946 (0.4) |  |  |
| **Social welfare the year prior to BZDR initiation** |  |  |  |  |
| No | 327386 (93.6) | 218490 (90.6) | 1.00 | 1.00 |
| Yes | 21593 (6.2) | 22465 (9.3) | **1.40 (1.39-1.42)** | **1.06 (1.05-1.08)** |
| Missing / unclear | 641 (0.2) | 145 (0.1) |  |  |
| **Unemployment the year prior to BZDR initiation** |  |  |  |  |
| No | 321207 (91.9) | 220514 (91.4) | 1.00 | 1.00 |
| Yes | 27772 (7.9) | 20441 (8.5) | **1.04 (1.03-1.06)** | 1.00 (0.99-1.02) |
| Missing / unclear / under age 19 the year prior to BZDR initiation^b^ | 641 (0.2) | 145 (0.1) |  |  |
| **Disability pension the year prior to BZDR initiation** |  |  |  |  |
| No | 310886 (88.9) | 197068 (81.7) | 1.00 | 1.00 |
| Yes | 38093 (10.9) | 43887 (18.2) | **1.50 (1.49-1.52)** | **1.21 (1.19-1.22)** |
| Missing / unclear / under age 19 the year prior to BZDR initiation^b^ | 641 (0.2) | 2999 (0.1) |  |  |

*Note*: Significant hazard rations are written in bold. Inverse associations are written in Italics. The analyses are conducted among the complete analytical sample, i.e., among individuals with all covariates available.

^a^ Stockholm County – the largest county in Sweden, Skåne – the second largest county in the country, Västra Götaland – the third largest county in Sweden.

^b^ In Sweden, unemployment benefits and disability pension benefits are paid from age 19 years to 65 years.

**Supplementary Table S7**. Hazard ratios (HR) and 95% confidence intervals (95% CI) for long-term BZDR use versus short-term use in associations with demographic, clinical, pharmacological, and socio-economic characteristics in age group of 65 years and older at BZDR initiation

|  | **Short-term users**  **n=138,493** | **Long-term users**  **n=182,768** | **Minimally-adjusted for sex** | **Fully-adjusted for all covariates** |
| --- | --- | --- | --- | --- |
|  | **n (%)** | **n (%)** | **HR (95% CI)** | **HR (95% CI)** |
| **Sex** |  |  |  |  |
| Male | 58332 (42.1) | 73516 (40.2) | 1.00 | 1.00 |
| Female | 80161 (58.9) | 109252 (59.8) | 1.02 (1.01-1.03) | 0.99 (0.98-1.00) |
| **Place of birth** |  |  |  |  |
| Sweden | 122272 (88.3) | 162818 (89.1) | 1.00 | 1.00 |
| Other countries | 16221 (11.7) | 19950 (10.9) | ***0.94 (0.93-0.96)*** | ***0.94 (0.92-0.95)*** |
| **Residence in Sweden at BZDR initiation^a^** |  |  |  |  |
| Stockholm county | 27076 (19.6) | 33143 (18.1) | 1.00 | 1.00 |
| Skåne | 18963 (13.7) | 24881 (13.6) | **1.05 (1.03-1.07)** | **1.04 (1.02-1.06)** |
| Västra Götaland | 22593 (16.3) | 31571 (17.3) | **1.09 (1.08-1.11)** | 1.00 (0.98-1.02) |
| Other county | 69204 (49.9) | 92494 (50.6) | **1.07 (1.06-1.08)** | ***0.98 (0.97-0.99)*** |
| Missing / unclear | 657 (0.5) | 679 (0.4) |  |  |
| **Psychiatric disorders ever before BZDR initiation** |  |  |  |  |
| Neuropsychiatric disorders | 64 (0.05) | 58 (0.03) | 0.80 (0.62-1.04) | ***0.75 (0.57-0.98)*** |
| Substance use disorders | 2807 (2.0) | 4080 (2.2) | **1.13 (1.09-1.16)** | 1.00 (0.96-1.03) |
| Affective disorders | 4245 (3.1) | 7332 (4.0) | **1.23 (1.20-1.26)** | ***0.92 (0.89-0.94)*** |
| Anxiety and other neurotic, stress-related and somatoform disorders | 3211 (2.3) | 4524 (2.5) | **1.06 (1.03-1.10)** | 0.97 (0.94-1.00) |
| Schizophrenia and psychotic disorders | 1006 (0.7) | 1573 (0.9) | **1.18 (1.12-1.24)** | ***0.86 (0.81-0.90)*** |
| Mental retardation and conduct disorders | 102 (0.1) | 124 (0.1) | 1.03 (0.86-1.22) | 0.85 (0.71-1.01) |
| **Somatic disorders ever before BZDR initiation** |  |  |  |  |
| None or 1 somatic disorder | 95312 (68.8) | 118213 (64.7) | 1.00 | 1.00 |
| Somatic multimorbidity (2 or more disorders) | 43181 (31.2) | 64555 (35.3) | **1.21 (1.20-1.22)** | **1.10 (1.09-1.11)** |
| **Type of BZDR used at initiation** |  |  |  |  |
| Anxiolytic | 53465 (38.6) | 36610 (20.0) | 1.00 | 1.00 |
| Hypnotic/sedative | 2127 (1.5) | 2453 (1.3) | **1.38 (1.33-1.44)** | **1.51 (1.45-1.58)** |
| Antiepileptic | 509 (0.4) | 306 (0.2) | 0.90 (0.80-1.00) | 0.97 (0.86-1.08) |
| Z-drug | 67181 (48.5) | 77448 (42.4) | **1.54 (1.52-1.56)** | **1.74 (1.72-1.76)** |
| Multiple BZDRs at initiation | 15211 (11.0) | 65951 (36.1) | **3.29 (3.24-3.33)** | **3.29 (3.25-3.34)** |
| **Medication dispensed ≤3 months before BZDR initiation** |  |  |  |  |
| Antidepressants | 38784 (28.0) | 83300 (45.6) | **1.62 (1.61-1.64)** | **1.43 (1.41-1.44)** |
| Psychostimulants | 97 (0.1) | 176 (0.1) | **1.22 (1.05-1.42)** | 1.11 (0.95-1.30) |
| Mood stabilisers | 2324 (1.7) | 4718 (2.6) | **1.31 (1.28-1.35)** | **1.13 (1.10-1.17)** |
| (Non-BZDR)-antiepileptics | 6804 (4.9) | 14972 (8.2) | **1.39 (1.36-1.41)** | **1.13 (1.11-1.15)** |
| Antipsychotics | 8776 (6.3) | 27281 (14.9) | **1.80 (1.78-1.83)** | **1.44 (1.42-1.46)** |
| (Non-BZDR)-anxiolytics/hypnotics/sedatives | 25177 (18.2) | 59700 (32.7) | **1.59 (1.58-1.61)** | **1.34 (1.32-1.35)** |
| Analgesics | 81171 (58.6) | 130924 (71.6) | **1.44 (1.43-1.46)** | **1.19 (1.17-1.20)** |
| Opioids | 61013 (44.0) | 102542 (56.1) | **1.34 (1.32-1.35)** | **1.14 (1.13-1.15)** |
| **Prescriber care level at BZDR initiation** |  |  |  |  |
| Psychiatry care | 7874 (5.7) | 12321 (6.7) | 1.00 | 1.00 |
| Primary care | 96601 (69.8) | 128610 (70.4) | ***0.90 (0.88-0.92)*** | **1.05 (1.03-1.07)** |
| Specialized non-psychiatric care | 339728 (24.5) | 41713 (22.8) | ***0.88 (0.86-0.89)*** | 0.99 (0.97-1.02) |
| Multiple prescribers | 42 (0.03) | 108 (0.06) | **1.48 (1.23-1.79)** | **1.25 (1.03-1.51)** |
| Missing / unclear | ≤10 | 16 (0.01) |  |  |
| **Disposable income the year prior to BZDR initiation** |  |  |  |  |
| Lowest quartile | 31095 (22.4) | 46607 (25.5) | 1.00 | 1.00 |
| 2^nd^ quartile | 40690 (29.4) | 60322 (33.0) | 1.02 (1.00-1.03) | 0.99 (0.98-1.01) |
| 3^d^ quartile | 35173 (25.4) | 44703 (24.5) | ***0.92 (0.91-0.93)*** | ***0.95 (0.94-0.96)*** |
| Highest quartile | 31105 (22.5) | 30871 (16.9) | ***0.77 (0.76-0.78)*** | ***0.85 (0.84-0.86)*** |
| Missing / unclear | 430 (0.3) | 265 (0.1) |  |  |

*Note*: Significant hazard rations are written in bold. Inverse associations are written in Italics. The analyses are conducted among the complete analytical sample, i.e., among the individuals with all data on covariates available. Covariates were removed from the analyses if: 1) data in the register were incomplete with a substantial proportion of missing values (in the age group of 65 years and older: civil status); 2) characteristics are not relevant for age group (e.g., unemployment, disability pension and social welfare for individuals aged 65 and older, i.e., above the age of retirement). If a certain characteristic is reported only in 10 or less individuals, the actual frequencies are replaced with ‘≤10’ to mitigate any possibility for backward identification.

^a^ Stockholm County – the largest county in Sweden, Skåne – the second largest county in the country, Västra Götaland – the third largest county in Sweden

**Supplementary Table S8**. Trajectory groups of BZDR use and odds ratio adjusted for sex among individuals aged 0-17 years at the initiation of treatment (n=5065).

|  | **“Discontinued” trajectory (n=3798)** | **“Decreasing” trajectory**  **(n=420)** | **“Decreasing” versus “discontinued” (ref)** | **“Slow decreasing” trajectory (n=614)** | **“Slow decreasing” versus “discontinued” (ref)** | **“Maintained” trajectory (n=233)** | **“Maintained” versus “discontinued” (ref)** |
| --- | --- | --- | --- | --- | --- | --- | --- |
|  | **n (%)** | **n (%)** | **OR (95% CI) sex-adj.** | **n (%)** | **OR (95% CI) sex-adj.** | **n (%)** | **OR (95% CI) sex-adj.** |
| **Sex** |  |  |  |  |  |  |  |
| Male | 1824 (48.0) | 170 (40.5) | 1.00 | 244 (39.7) | 1.00 | 75 (32.2) | 1.00 |
| Female | 1974 (52.0) | 250 (59.5) | **1.28 (1.04-1.59)** | 370 (60.3) | **1.36 (1.14-1.62)** | 158 (67.8) | **1.90 (1.41-2.57)** |
| **Place of birth** |  |  |  |  |  |  |  |
| Sweden | 3703 (97.5) | 399 (95.0) | 1.00 | 601 (97.9) | 1.00 | 226 (97.0) | 1.00 |
| Other countries | 95 (2.5) | 21 (5.0) | 2.03 (0.98-4.22) | 13 (2.1) | 0.60 (0.21-1.68) | ≤10 | 0.41 (0.06-3.03) |
| **Residence in Sweden at BZDR initiation** |  |  |  |  |  |  |  |
| Stockholm county | 815 (21.5) | 93 (22.1) | 1.00 | 136 (22.1) | 1.00 | 43 (18.4) | 1.00 |
| Skåne | 386 (10.2) | 39 (9.3) | 0.85 (0.56-1.29) | 57 (9.3) | 0.87 (0.61-1.22) | 21 (9.0) | 1.13 (0.65-1.97) |
| Västra Götaland | 572 (15.1) | 53 (12.6) | 0.79 (0.55-1.15) | 93 (15.2) | 0.90 (0.67-1.21) | 50 (51.5) | **1.61 (1.02-2.52)** |
| Other county | 1988 (52.3) | 231 (55.0) | 1.00 (0.77-1.29) | 323 (52.6) | 0.95 (0.76-1.18) | 119 (51.1) | 1.09 (0.75-1.60) |
| Missing / unclear | 37 (0.9) | ≤10 |  | ≤10 |  | 0 |  |
| **Psychiatric disorders ever before BZDR initiation** |  |  |  |  |  |  |  |
| Neuropsychiatric disorders | 234 (6.2) | 48 (11.4) | **2.35 (1.67-3.31)** | 83 (13.5) | **2.58 (1.94-3.44)** | 45 (19.3) | **4.65 (3.18-6.81)** |
| Substance use disorders | 84 (2.2) | 16 (3.81) | **2.00 (1.15-3.48)** | 37 (6.0) | **2.73 (1.79-4.19)** | 21 (9.0) | **4.30 (2.51-7.37)** |
| Affective disorders | 303 (8.0) | 74 (17.6) | **2.55 (1.90-3.41)** | 110 (17.9) | **2.53 (1.97-3.25)** | 84 (36.1) | **6.75 (4.94-9.23)** |
| Anxiety and other neurotic, stress-related and somatoform disorders | 276 (7.3) | 75 (17.9) | **2.82 (2.09-3.79)** | 116 (18.9) | **3.14 (2.45-4.02)** | 75 (32.2) | **5.56 (4.00-7.72)** |
| Schizophrenia and psychotic disorders | 17 (0.4) | ≤10 | **4.48 (1.81-11.1)** | ≤10 | **3.47 (1.46-8.23)** | ≤10 | **10.3 (4.29-24.8)** |
| Mental retardation and conduct disorders | 99 (2.6) | 27 (6.43) | **3.15 (2.01-4.92)** | 38 (6.2) | **2.67 (1.77-4.01)** | 23 (9.9) | **4.79 (2.87-8.00)** |
| **Somatic disorders ever before BZDR initiation** |  |  |  |  |  |  |  |
| None or 1 somatic disorder | 3780 (99.5) | 418 (99.5) | 1.00 | 611 (99.5) | 1.00 | 231 (99.1) | 1.00 |
| Somatic multimorbidity (2 or more disorders) | 18 (0.5) | ≤10 | 1.16 (0.27-5.07) | ≤10 | 1.18 (0.34-4.06) | ≤10 | 2.22 (0.51-9.79) |
| **Type of BZDR used at initiation** |  |  |  |  |  |  |  |
| Anxiolytic | 2599 (68.4) | 171 (40.7) | 1.00 | 249 (40.6) | 1.00 | 59 (25.3) | 1.00 |
| Hypnotic/sedative | 36 (0.9) | 11 (2.62) | **5.10 (2.53-10.3)** | ≤10 | **2.31 (1.01-5.27)** | ≤10 | 1.50 (0.20-11.2) |
| Antiepileptic | 23 (0.6) | ≤10 | **3.54 (1.32-9.48)** | ≤10 | 2.02 (0.69-5.92) | ≤10 | **7.16 (2.07-24.8)** |
| Z-drug | 942 (24.8) | 182 (43.3) | **2.95 (2.33-3.71)** | 287 (46.7) | **3.46 (2.85-4.20)** | 130 (55.8) | **6.45 (4.59-9.07)** |
| Multiple BZDRs at initiation | 198 (5.2) | 51 (12.1) | **3.74 (2.60-5.39)** | 66 (10.8) | **3.64 (2.65-5.02)** | 40 (17.2) | **9.23 (5.86-14.6)** |
| **Medication dispensed ≤3 months before BZDR initiation** |  |  |  |  |  |  |  |
| Antidepressants | 951 (25.0) | 254 (60.5) | **4.65 (3.72-5.80)** | 416 (67.7) | **6.68 (5.50-8.12)** | 215 (92.3) | **37.0 (22.0-62.2)** |
| Psychostimulants | 354 (9.3) | 91 (21.7) | **2.93 (2.24-3.82)** | 167 (27.2) | **3.81 (3.06-4.74)** | 101 (43.3) | **7.91 (5.85-10.7)** |
| Mood stabilisers | 187 (4.9) | 68 (16.2) | **3.80 (2.80-5.17)** | 177 (28.8) | **7.28 (5.74-9.24)** | 110 (47.2) | **15.3 (11.2-21.0)** |
| (Non-BZD)-antiepileptics | 92 (2.4) | 32 (7.6) | **3.07 (1.98-4.77)** | 84 (13.7) | **6.28 (4.56-8.64)** | 90 (38.6) | **24.7 (17.4-35.2)** |
| Antipsychotics | 285 (7.5) | 127 (30.2) | **5.60 (4.35-7.21)** | 237 (38.6) | **7.91 (6.40-9.79)** | 158 (67.8) | **26.2 (19.1-36.1)** |
| (Non-BZD)-anxiolytics/hypnotics/sedatives | 937 (24.7) | 262 (62.4) | **4.93 (3.96-6.14)** | 434 (70.7) | **7.58 (6.22-9.22)** | 218 (93.6) | **50.5 (28.0-91.0)** |
| Analgesics | 656 (17.3) | 107 (25.5) | **1.61 (1.26-2.06)** | 180 (29.3) | **1.92 (1.57-2.35)** | 102 (43.8) | **3.73 (2.79-4.99)** |
| Opioids | 479 (12.6) | 99 (23.6) | **2.22 (1.72-2.87)** | 190 (30.9) | **3.08 (2.51-3.79)** | 91 (39.1) | **4.05 (2.99-5.48)** |
| **Prescriber care level at BZDR initiation** |  |  |  |  |  |  |  |
| Psychiatry care | 1002 (26.4) | 209 (49.8) | 1.00 | 322 (52.4) | 1.00 | 162 (69.5) | 1.00 |
| Primary care | 872 (22.9) | 95 (22.6) | ***0.50 (0.38-0.66)*** | 147 (23.9) | ***0.52 (0.42-0.65)*** | 44 (18.9) | ***0.31 (0.21-0.44)*** |
| Specialized non-psychiatric care | 1924 (50.7) | 116 (27.6) | ***0.29 (0.23-0.37)*** | 145 (23.6) | ***0.22 (0.18-0.27)*** | 27 (11.6) | ***0.08 (0.05-0.13)*** |
| **Mother’s civil status the year prior to child’s BZDR initiation** |  |  |  |  |  |  |  |
| Married/cohabiting | 1857 (48.9) | 204 (48.6) | 1.00 | 279 (45.4) | 1.00 | 95 (40.8) | 1.00 |
| Unmarried | 1298 (34.2) | 113 (26.9) | ***0.78 (0.61-0.99)*** | 163 (26.6) | 0.86 (0.70-1.06) | 54 (23.2) | 0.82 (0.58-1.17) |
| Divorced/widow(er) | 529 (13.9) | 83 (19.8) | **1.46 (1.11-1.93)** | 148 (24.1) | **1.95 (1.56-2.45)** | 70 (30.0) | **2.51 (1.79-3.53)** |
| Missing / unclear | 114 (3.0) | 20 (4.76) |  | 24 (3.9) |  | 14 (6.0) |  |
| **Mother’s disposable income the year prior to child’s BZDR initiation** |  |  |  |  |  |  |  |
| Lowest quartile | 1662 (43.8) | 135 (32.1) | 1.00 | 194 (31.6) | 1.00 | 61 (26.2) | 1.00 |
| 2^nd^ quartile | 796 (20.9) | 106 (25.2) | **1.57 (1.20-2.07)** | 170 (27.7) | **1.83 (1.46-2.30)** | 57 (24.5) | **1.77 (1.20-2.61)** |
| 3^d^ quartile | 725 (19.1) | 91 (21.7) | **1.51 (1.14-2.02)** | 140 (22.8) | **1.64 (1.29-2.09)** | 58 (24.9) | **2.17 (1.49-3.17)** |
| Highest quartile | 522 (13.7) | 73 (17.4) | **1.65 (1.21-2.25)** | 92 (15.0) | **1.46 (1.11-1.91)** | 48 (20.6) | **2.08 (1.38-3.16)** |
| Missing / unclear | 93 (2.5) | 15 (3.6) |  | 18 (2.9) |  | ≤10 |  |
| **Mother’s social welfare the year prior to child’s BZDR initiation** |  |  |  |  |  |  |  |
| No | 3292 (86.7) | 334 (79.5) | 1.00 | 493 (80.3) | 1.00 | 184 (79.0) | 1.00 |
| Yes | 418 (11.0) | 71 (16.9) | **1.63 (1.22-2.17)** | 103 (16.8) | **1.64 (1.29-2.10)** | 40 (17.2) | **1.83 (1.26-2.64)** |
| Missing / unclear | 88 (2.3) | 15 (3.6) |  | 18 (2.9) |  | ≤10 |  |
| **Mother’s unemployment the year prior to child’s BZDR initiation** |  |  |  |  |  |  |  |
| No | 3241 (85.3) | 358 (85.2) | 1.00 | 536 (87.3) | 1.00 | 197 (84.5) | 1.00 |
| Yes | 469 (12.4) | 47 (11.2) | 0.91 (0.66-1.26) | 60 (9.8) | 0.75 (0.56-1.01) | 27 (11.6) | 1.01 (0.66-1.54) |
| Missing / unclear | 88 (2.3) | 15 (3.6) |  | 18 (2.9) |  | ≤10 |  |
| **Mother’s disability pension the year prior to child’s BZDR initiation** |  |  |  |  |  |  |  |
| No | 3339 (87.9) | 336 (80.0) | 1.00 | 479 (78.0) | 1.00 | 168 (72.1) | 1.00 |
| Yes | 371 (9.8) | 69 (16.4) | **1.76 (1.31-2.37)** | 117 (19.1) | **2.26 (1.79-2.86)** | 56 (24.0) | **2.91 (2.07-4.08)** |
| Missing / unclear | 88 (2.3) | 15 (3.6) |  | 18 (2.9) |  | ≤10 |  |
| **Father’s civil status the year prior to child’s BZDR initiation** |  |  |  |  |  |  |  |
| Married/cohabiting | 1846 (48.6) | 210 (50.0) | 1.00 | 271 (44.1) | 1.00 | 95 (40.8) | 1.00 |
| Unmarried | 1256 (33.1) | 109 (26.0) | ***0.77 (0.60-0.98)*** | 174 (28.3) | 0.93 (0.75-1.14) | 52 (22.3) | 0.84 (0.59-1.18) |
| Divorced/widower(er) | 506 (13.3) | 74 (17.6) | 1.26 (0.95-1.68) | 140 (22.8) | **1.83 (1.45-2.31)** | 64 (27.5) | **2.36 (1.68-3.32)** |
| Missing / unclear | 190 (5.0) | 27 (6.4) |  | 29 (4.7) |  | 22 (9.4) |  |
| **Father’s disposable income the year prior to child’s BZDR initiation** |  |  |  |  |  |  |  |
| Lowest quartile | 769 (20.2) | 59 (14.0) | 1.00 | 85 (13.8) | 1.00 | 28 (12.0) | 1.00 |
| 2^nd^ quartile | 656 (17.3) | 71 (16.9) | 1.40 (0.98-2.01) | 92 (15.0) | 1.26 (0.92-1.73) | 50 (21.5) | **1.93 (1.19-3.13)** |
| 3^rd^ quartile | 1091 (28.7) | 119 (28.3) | 1.37 (0.99-1.90) | 176 (28.7) | **1.42 (1.08-1.88)** | 58 (24.9) | 1.45 (0.92-2.31) |
| Highest quartile | 1089 (28.7) | 144 (34.3) | **1.70 (1.24-2.34)** | 230 (37.5) | **1.85 (1.42-2.42)** | 75 (32.2) | **1.77 (1.13-2.77)** |
| Missing / unclear | 193 (5.1) | 27 (6.4) |  | 31 (5.0) |  | 22 (9.4) |  |
| **Father’s social welfare the year prior to child’s BZDR initiation** |  |  |  |  |  |  |  |
| No | 3307 (87.1) | 349 (83.1) | 1.00 | 521 (84.8) | 1.00 | 185 (79.4) | 1.00 |
| Yes | 309 (8.1) | 45 (10.7) | 1.40 (1.00-1.96) | 65 (10.6) | 1.30 (0.97-1.74) | 27 (11.6) | 1.52 (0.98-2.35) |
| Missing / unclear | 182 (4.8) | 26 (6.2) |  | 28 (4.6) |  | 21 (9.0) |  |
| **Father’s unemployment the year prior to child’s BZDR initiation** |  |  |  |  |  |  |  |
| No | 3282 (86.4) | 354 (84.3) | 1.00 | 532 (86.6) | 1.00 | 189 (81.1) | 1.00 |
| Yes | 334 (8.8) | 40 (9.5) | 1.13 (0.80-1.59) | 54 (8.8) | 1.00 (0.73-1.35) | 23 (9.9) | 1.24 (0.79-1.94) |
| Missing / unclear | 182 (4.8) | 26 (6.2) |  | 28 (4.6) |  | 21 (9.0) |  |
| **Father’s disability pension the year prior to child’s BZDR initiation** |  |  |  |  |  |  |  |
| No | 3406 (89.7) | 352 (83.8) | 1.00 | 536 (87.3) | 1.00 | 185 (79.4) | 1.00 |
| Yes | 210 (5.5) | 42 (10.0) | **1.96 (1.38-2.79)** | 50 (8.1) | **1.51 (1.09-2.10)** | 27 (11.6) | **2.38 (1.54-3.68)** |
| Missing / unclear | 182 (4.8) | 26 (6.2) |  | 28 (4.6) |  | 21 (9.0) |  |

*Note:* Significant estimates are written in bold. Inverse associations are written in *Italics*. The analyses are conducted among the complete analytical sample, i.e., among the individuals with all data on covariates available. If a certain characteristic is reported only in 10 or less individuals, the actual frequencies are replaced with ‘≤10’ to mitigate any possibility for backward identification. BZDR, benzodiazepines and benzodiazepine-related drugs; CI, confidence intervals; OR, odds ratio

**Supplementary Table S9**. Trajectory groups of BZDR use and odds ratio adjusted for sex among individuals aged 18-64 years at the initiation of treatment (n=219,316).

|  | **“Discontinued” trajectory (n=111,959)** | **“Decreasing” trajectory**  **(n=24,707)** | **“Decreasing” versus “discontinued” (ref)** | **“Slow decreasing” trajectory (n=66,625)** | **“Slow decreasing” versus “discontinued” (ref)** | **“Maintained” trajectory (n=16,025)** | **“Maintained” versus “discontinued” (ref)** |
| --- | --- | --- | --- | --- | --- | --- | --- |
|  | **n (%)** | **n (%)** | **OR (95% CI) sex-adj.** | **n (%)** | **OR (95% CI) sex-adj.** | **n (%)** | **OR (95% CI) sex-adj.** |
| **Sex** |  |  |  |  |  |  |  |
| Male | 47040 (42.0) | 9870 (39.9) | 1.00 | 24265 (36.42) | 1.00 | 6491 (40.5) | 1.00 |
| Female | 64919 (58.0) | 14837 (60.1) | **1.08 (1.05-1.11)** | 42360 (63.58) | **1.26 (1.24-1.29)** | 9534 (59.5) | **1.06 (1.02-1.09)** |
| **Place of birth** |  |  |  |  |  |  |  |
| Sweden | 91856 (82.0) | 19978 (80.9) | 1.00 | 55075 (82.66) | 1.00 | 13557 (84.6) | 1.00 |
| Other countries | 20103 (18.0) | 4729 (19.1) | **1.08 (1.05-1.12)** | 11550 (17.34) | ***0.96 (0.94-0.99)*** | 2468 (15.4) | ***0.84 (0.80-0.88)*** |
| **Residence in Sweden at BZDR initiation** |  |  |  |  |  |  |  |
| Stockholm county | 27656 (24.7) | 6466 (26.1) | 1.00 | 17411 (26.13) | 1.00 | 3371 (21.0) | 1.00 |
| Skåne | 14055 (12.5) | 3203 (13.0) | 0.97 (0.93-1.02) | 9003 (13.51) | 1.02 (0.98-1.05) | 2052 (12.8) | **1.21 (1.14-1.28)** |
| Västra Götaland | 19872 (17.8) | 4491 (18.2) | 0.97 (0.93-1.01) | 12468 (18.71) | 1.00 (0.97-1.03) | 3053 (19.1) | **1.27 (1.21-1.34)** |
| Other county | 49833 (44.5) | 10525 (42.6) | ***0.90 (0.87-0.93)*** | 27702 (41.58) | ***0.88 (0.86-0.91)*** | 7548 (47.1) | **1.25 (1.19-1.30)** |
| Missing / unclear | 543 (0.5) | 22 (0.1) |  | 41 (0.06) |  | ≤10 |  |
| **Psychiatric disorders ever before BZDR initiation** |  |  |  |  |  |  |  |
| Neuropsychiatric disorders | 758 (0.7) | 297 (1.2) | **1.82 (1.59-2.08)** | 741 (1.11) | **1.74 (1.57-1.93)** | 305 (1.9) | **2.90 (2.54-3.32)** |
| Substance use disorders | 5220 (4.7) | 1726 (7.0) | **1.59 (1.50-1.68)** | 4116 (6.18) | **1.45 (1.38-1.51)** | 1907 (11.9) | **2.87 (2.71-3.03)** |
| Affective disorders | 8935 (8.0) | 2762 (11.2) | **1.45 (1.39-1.52)** | 7132 (10.70) | **1.39 (1.34-1.43)** | 2352 (14.7) | **1.98 (1.89-2.08)** |
| Anxiety and other neurotic, stress-related and somatoform disorders | 10728 (9.6) | 3070 (12.4) | **1.34 (1.28-1.40)** | 7773 (11.67) | **1.25 (1.21-1.29)** | 2520 (15.7) | **1.76 (1.68-1.85)** |
| Schizophrenia and psychotic disorders | 1583 (1.4) | 604 (2.4) | **1.79 (1.63-1.97)** | 1592 (2.39) | **1.79 (1.67-1.92)** | 788 (4.9) | **3.67 (3.36-4.01)** |
| Mental retardation and conduct disorders | 302 (0.3) | 96 (0.4) | **1.46 (1.16-1.84)** | 307 (0.46) | **1.77 (1.51-2.07)** | 122 (0.8) | **2.87 (2.32-3.54)** |
| **Somatic disorders ever before BZDR initiation** |  |  |  |  |  |  |  |
| None or 1 somatic disorder | 107477 (96.0) | 23446 (94.9) | 1.00 | 63341 (95.07) | 1.00 | 14799 (92.4) | 1.00 |
| Somatic multimorbidity (2 or more disorders) | 4482 (4.0) | 1261 (5.1) | **1.30 (1.22-1.39)** | 3284 (4.93) | **1.29 (1.23-1.36)** | 1226 (7.6) | **2.02 (1.89-2.16)** |
| **Type of BZDR used at initiation** |  |  |  |  |  |  |  |
| Anxiolytic | 36778 (32.8) | 6392 (25.9) | 1.00 | 17008 (25.53) | 1.00 | 2776 (17.3) | 1.00 |
| Hypnotic/sedative | 1672 (1.5) | 404 (1.6) | **1.40 (1.25-1.57)** | 1160 (1.74) | **1.53 (1.42-1.66)** | 223 (1.4) | **1.75 (1.51-2.03)** |
| Antiepileptic | 354 (0.3) | 95 (0.4) | **1.57 (1.24-1.98)** | 209 (0.31) | **1.34 (1.13-1.60)** | 61 (0.4) | **2.37 (1.80-3.13)** |
| Z-drug | 58741 (52.5) | 12391 (50.1) | **1.21 (1.17-1.26)** | 33832 (50.78) | **1.25 (1.22-1.28)** | 7702 (48.1) | **1.72 (1.65-1.80)** |
| Multiple BZDRs at initiation | 14414 (12.9) | 5425 (22.0) | **2.18 (2.09-2.27)** | 14416 (21.64) | **2.18 (2.12-2.25)** | 5263 (32.8) | **4.83 (4.59-5.08)** |
| **Medication dispensed ≤3 months before BZDR initiation** |  |  |  |  |  |  |  |
| Antidepressants | 52953 (47.3) | 15371 (62.2) | **1.83 (1.78-1.88)** | 41511 (62.31) | **1.82 (1.79-1.86)** | 11764 (73.4) | **3.08 (2.96-3.20)** |
| Psychostimulants | 1830 (1.6) | 863 (3.5) | **2.20 (2.03-2.39)** | 2748 (4.12) | **2.66 (2.51-2.83)** | 1197 (7.5) | **4.90 (4.55-5.28)** |
| Mood stabilisers | 2828 (2.5) | 1402 (5.7) | **2.35 (2.20-2.51)** | 4465 (6.70) | **2.82 (2.69-2.96)** | 1930 (12.0) | **5.33 (5.02-5.67)** |
| (Non-BZD)-antiepileptics | 6661 (5.9) | 2781 (11.3) | **2.02 (1.93-2.12)** | 8061 (12.10) | **2.21 (2.13-2.28)** | 3534 (22.1) | **4.52 (4.32-4.73)** |
| Antipsychotics | 6237 (5.6) | 3063 (12.4) | **2.44 (2.33-2.56)** | 8892 (13.35) | **2.71 (2.62-2.80)** | 4211 (26.3) | **6.19 (5.93-6.47)** |
| (Non-BZD)-anxiolytics/hypnotics/sedatives | 37939 (33.9) | 12335 (49.9) | **1.94 (1.89-2.00)** | 34068 (51.13) | **2.04 (2.00-2.08)** | 10482 (65.4) | **3.70 (3.57-3.83)** |
| Analgesics | 48635 (43.4) | 12512 (50.6) | **1.33 (1.30-1.37)** | 35116 (52.71) | **1.43 (1.40-1.46)** | 9845 (61.4) | **2.08 (2.01-2.15)** |
| Opioids | 47686 (42.6) | 12487 (50.5) | **1.38 (1.34-1.42)** | 34377 (51.60) | **1.44 (1.41-1.47)** | 9638 (60.1) | **2.04 (1.97-2.11)** |
| **Prescriber care level at BZDR initiation** |  |  |  |  |  |  |  |
| Psychiatry care | 19593 (17.5) | 5165 (20.9) | 1.00 | 12995 (19.50) | 1.00 | 3911 (24.4) | 1.00 |
| Primary care | 72737 (65.0) | 15319 (62.0) | ***0.79 (0.77-0.82)*** | 42928 (64.43) | ***0.87 (0.85-0.90)*** | 9850 (61.5) | ***0.67 (0.65-0.70)*** |
| Specialized care | 19584 (17.5) | 4210 (17.0) | ***0.81 (0.78-0.85)*** | 10653 (15.99) | ***0.82 (0.79-0.85)*** | 2249 (14.0) | ***0.58 (0.55-0.61)*** |
| Multiple prescribers | 37 (0.03) | 9 (0.04) | 0.95 (0.46-1.97) | 44 (0.07) | **1.69 (1.07-2.65)** | 12 (0.1) | 1.67 (0.87-3.22) |
| Missing / unclear | ≤10 | ≤10 |  | ≤10 |  | ≤10 |  |
| **Civil status the year before BZDR initiation** |  |  |  |  |  |  |  |
| Married/register partner | 48176 (43.0) | 10421 (42.2) | 1.00 | 29686 (44.56) | 1.00 | 6169 (38.5) | 1.00 |
| Unmarried | 45243 (40.4) | 9667 (39.1) | 0.99 (0.96-1.02) | 24290 (36.46) | ***0.89 (0.87-0.91)*** | 6364 (39.7) | **1.10 (1.06-1.14)** |
| Divorced/widow(er) | 16689 (14.9) | 4208 (17.0) | **1.16 (1.12-1.21)** | 11494 (17.25) | **1.11 (1.08-1.14)** | 3235 (20.2) | **1.51 (1.45-1.58)** |
| Missing / unclear | 1851 (1.7) | 411 (1.7) |  | 1155 (1.73) |  | 257 (1.6) |  |
| **Disposable income the year before BZDR initiation** |  |  |  |  |  |  |  |
| Lowest quartile | 31063 (27.7) | 7069 (28.6) | 1.00 | 17991 (27.00) | 1.00 | 4870 (30.4) | 1.00 |
| 2^nd^ quartile | 24783 (22.1) | 5708 (23.1) | 1.02 (0.98-1.06) | 15582 (23.39) | **1.09 (1.06-1.12)** | 4464 (27.9) | **1.15 (1.10-1.20)** |
| 3^rd^ quartile | 29061 (26.0) | 6051 (24.5) | ***0.92 (0.89-0.96)*** | 16823 (25.25) | 1.03 (1.00-1.06) | 3760 (23.5) | ***0.82 (0.78-0.86)*** |
| Highest quartile | 26383 (23.6) | 5783 (23.4) | 0.98 (0.94-1.02) | 15996 (24.01) | **1.11 (1.08-1.14)** | 2887 (18.0) | ***0.69 (0.65-0.72)*** |
| Missing / unclear | 669 (0.6) | 96 (0.4) |  | 233 (0.35) |  | 44 (0.3) |  |
| **Social welfare the year before BZDR initiation** |  |  |  |  |  |  |  |
| No | 104067 (93.0) | 22539 (91.2) | 1.00 | 61701 (92.61) | 1.00 | 14213 (88.7) | 1.00 |
| Yes | 7556 (6.7) | 2143 (8.7) | **1.31 (1.24-1.38)** | 4881 (7.33) | **1.10 (1.06-1.15)** | 1811 (11.3) | **1.78 (1.68-1.88)** |
| Missing / unclear | 336 (0.3) | 25 (0.1) |  | 43 (0.06) |  | ≤10 |  |
| **Unemployment the year before BZDR initiation** |  |  |  |  |  |  |  |
| No | 100482 (89.7) | 22255 (90.1) | 1.00 | 60288 (90.49) | 1.00 | 14458 (90.2) | 1.00 |
| Yes | 11141 (10.0) | 2427 (9.8) | 0.98 (0.94-1.03) | 6294 (9.45) | ***0.93 (0.90-0.96)*** | 1566 (9.8) | 0.98 (0.92-1.03) |
| Missing / unclear | 336 (0.3) | 25 (0.1) |  | 43 (0.06) |  | ≤10 |  |
| **Disability pension the year before BZDR initiation** |  |  |  |  |  |  |  |
| No | 97030 (86.7) | 20186 (81.7) | 1.00 | 53532 (80.35) | 1.00 | 11170 (69.7) | 1.00 |
| Yes | 14593 (13.0) | 4496 (18.2) | **1.49 (1.43-1.54)** | 13050 (19.59) | **1.62 (1.57-1.66)** | 4854 (30.3) | **2.93 (2.82-3.05)** |
| Missing / unclear | 336 (0.3) | 25 (0.1) |  | 43 (0.06) |  | ≤10 |  |

*Note:* Significant estimates are written in bold. Inverse associations are written in Italics. The analyses are conducted among the complete analytical sample, i.e., among the individuals with all data on covariates available. If a certain characteristic is reported only in 10 or less individuals, the actual frequencies are replaced with ‘≤10’ to mitigate any possibility for backward identification.

* In Sweden, unemployment benefits and disability pension benefits are paid from age 19 years to 65 years.

*Abbreviation*: BZDR, benzodiazepines and benzodiazepine-related drugs; CI, confidence intervals; OR, odds ratio

**Supplementary Table S10**. Trajectory groups of BZDR use and odds ratio adjusted for sex among individuals aged 65 years and older at the initiation of treatment (n=113,418).

|  | **“Discontinued” trajectory (n=44,525)** | **“Decreasing” trajectory**  **(n=16,220)** | **“Decreasing” versus “discontinued” (ref)** | **“Slow decreasing” trajectory (n=11,014)** | **“Slow decreasing” versus “discontinued” (ref)** | **“Maintained” trajectory (n=41,659)** | **“Maintained” versus “discontinued” (ref)** |
| --- | --- | --- | --- | --- | --- | --- | --- |
|  | **n (%)** | **n (%)** | **OR (95% CI) sex-adj.** | **n (%)** | **OR (95% CI) sex-adj.** | **n (%)** | **OR (95% CI) sex-adj.** |
| **Sex** |  |  |  |  |  |  |  |
| Male | 18814 (42.2) | 6843 (42.2) | 1.00 | 4152 (37.7) | 1.00 | 14194 (34.1) | 1.00 |
| Female | 25711 (57.8) | 9377 (57.8) | 0.99 (0.96-1.03) | 6862 (62.3) | **1.20 (1.15-1.25)** | 27465 (65.9) | **1.40 (1.36-1.44)** |
| **Place of birth** |  |  |  |  |  |  |  |
| Sweden | 39405 (88.5) | 14459 (89.1) | 1.00 | 9764 (88.6) | 1.00 | 36977 (88.8) | 1.00 |
| Other countries | 5120 (11.5) | 1761 (10.9) | ***0.93 (0.88-0.99)*** | 1250 (11.4) | 0.98 (0.92-1.04) | 4682 (11.2) | 0.96 (0.92-1.00) |
| **Residence in Sweden at BZDR initiation** |  |  |  |  |  |  |  |
| Stockholm county | 8155 (18.3) | 3045 (18.8) | 1.00 | 2133 (19.4) | 1.00 | 8129 (19.5) | 1.00 |
| Skåne | 5669 (12.7) | 2163 (13.3) | 1.02 (0.96-1.09) | 1556 (14.1) | 1.05 (0.98-1.13) | 5862 (14.1) | 1.04 (0.99-1.09) |
| Västra Götaland | 7418 (16.7) | 2825 (17.4) | 1.02 (0.96-1.08) | 1890 (17.2) | 0.98 (0.91-1.05) | 7308 (17.5) | 1.00 (0.95-1.04) |
| Other county | 22150 (49.8) | 8171 (50.4) | 0.99 (0.94-1.04) | 5421 (49.2) | ***0.94 (0.89-0.99)*** | 20351 (48.9) | ***0.93 (0.89-0.96)*** |
| Missing / unclear | 1133 (2.5) | 16 (0.1) |  | 14 (0.1) |  | ≤10 |  |
| **Psychiatric disorders ever before BZDR initiation** |  |  |  |  |  |  |  |
| Neuropsychiatric disorders | 10 (0.02) | ≤10 | 1.07 (0.34-3.42) | ≤10 | 1.54 (0.48-4.90) | ≤10 | 0.39 (0.12-1.26) |
| Substance use disorders | 778 (1.8) | 292 (1.8) | 1.04 (0.90-1.19) | 160 (1.5) | 0.89 (0.75-1.05) | 733 (1.8) | **1.14 (1.03-1.26)** |
| Affective disorders | 1507 (3.4) | 667 (4.1) | **1.24 (1.12-1.36)** | 421 (3.8) | **1.15 (1.03-1.28)** | 1418 (3.4) | 1.01 (0.94-1.09) |
| Anxiety and other neurotic, stress-related and somatoform disorders | 821 (1.8) | 370 (2.3) | **1.23 (1.09-1.40)** | 266 (2.4) | **1.29 (1.12-1.49)** | 935 (2.2) | **1.19 (1.08-1.31)** |
| Schizophrenia and psychotic disorders | 337 (0.8) | 133 (0.8) | 1.10 (0.90-1.34) | 86 (0.8) | 1.04 (0.82-1.32) | 280 (0.7) | 0.89 (0.75-1.04) |
| Mental retardation and conduct disorders | 29 (0.1) | ≤10 | 0.92 (0.45-1.89) | ≤10 | 0.70 (0.27-1.82) | 15 (0.04) | 0.57 (0.31-1.07) |
| **Somatic disorders ever before BZDR initiation** |  |  |  |  |  |  |  |
| None or 1 somatic disorder | 30577 (68.7) | 10398 (64.1) | 1.00 | 7771 (70.6) | 1.00 | 31421 (75.4) | 1.00 |
| Somatic multimorbidity (2 or more disorders) | 13948 (31.3) | 5822 (35.9) | **1.25 (1.20-1.30)** | 3243 (29.4) | ***0.95 (0.91-0.99)*** | 10238 (24.6) | ***0.73 (0.71-0.76)*** |
| **Type of BZDR used at initiation** |  |  |  |  |  |  |  |
| Anxiolytic | 15261 (34.3) | 4488 (27.7) | 1.00 | 3028 (27.5) | 1.00 | 8219 (19.7) | 1.00 |
| Hypnotic/sedative | 801 (1.8) | 342 (2.1) | **1.46 (1.28-1.67)** | 290 (2.6) | **1.88 (1.64-2.17)** | 994 (2.4) | **2.41 (2.18-2.65)** |
| Antiepileptic | 133 (0.3) | 26 (0.2) | ***0.63 (0.41-0.97)*** | 19 (0.2) | 0.72 (0.45-1.17) | 76 (0.2) | 1.08 (0.81-1.43) |
| Z-drug | 19517 (43.8) | 6328 (39.0) | **1.10 (1.05-1.15)** | 4442 (40.3) | **1.16 (1.10-1.22)** | 18813 (45.2) | **1.83 (1.77-1.89)** |
| Multiple BZDRs at initiation | 8813 (19.8) | 5036 (31.0) | **1.98 (1.88-2.07)** | 3235 (29.4) | **1.90 (1.80-2.01)** | 13557 (32.5) | **2.95 (2.84-3.07)** |
| **Medication dispensed ≤3 months before BZDR initiation** |  |  |  |  |  |  |  |
| Antidepressants | 15677 (35.2) | 7673 (47.3) | **1.66 (1.60-1.72)** | 5060 (45.9) | **1.56 (1.49-1.63)** | 19576 (47.0) | **1.61 (1.57-1.66)** |
| Psychostimulants | 29 (0.1) | 14 (0.1) | 1.20 (0.62-2.31) | 12 (0.1) | 1.65 (0.84-3.23) | 46 (0.1) | **1.68 (1.06-2.68)** |
| Mood stabilisers | 772 (1.7) | 364 (2.2) | **1.30 (1.14-1.47)** | 238 (2.2) | **1.27 (1.09-1.47)** | 768 (1.8) | 1.09 (0.98-1.20) |
| (Non-BZD)-antiepileptics | 2695 (6.1) | 1259 (7.8) | **1.30 (1.21-1.39)** | 897 (8.1) | **1.37 (1.27-1.48)** | 3716 (8.9) | **1.52 (1.44-1.60)** |
| Antipsychotics | 5146 (11.6) | 3038 (18.7) | **1.82 (1.73-1.91)** | 1655 (15.0) | **1.40 (1.31-1.48)** | 4851 (11.6) | 1.04 (1.00-1.09) |
| (Non-BZD)-anxiolytics/hypnotics/sedatives | 11242 (25.3) | 5627 (34.7) | **1.58 (1.51-1.64)** | 3649 (33.1) | **1.48 (1.42-1.55)** | 14608 (35.1) | **1.62 (1.57-1.67)** |
| Analgesics | 30824 (69.2) | 12470 (76.9) | **1.48 (1.42-1.55)** | 8254 (74.9) | **1.32 (1.26-1.38)** | 30843 (74.0) | **1.24 (1.20-1.28)** |
| Opioids | 24695 (55.5) | 10163 (62.7) | **1.35 (1.30-1.40)** | 6655 (60.4) | **1.23 (1.18-1.28)** | 25172 (60.4) | **1.22 (1.19-1.26)** |
| **Prescriber care level at BZDR initiation** |  |  |  |  |  |  |  |
| Psychiatry care | 3316 (7.5) | 1206 (7.4) | 1.00 | 823 (7.5) | 1.00 | 2835 (6.8) | 1.00 |
| Primary care | 29487 (66.2) | 11061 (68.2) | 1.03 (0.96-1.11) | 7733 (70.2) | 1.05 (0.97-1.14) | 30684 (73.7) | **1.20 (1.14-1.27)** |
| Specialized care | 11700 (26.3) | 3942 (24.3) | 0.93 (0.86-1.01) | 2451 (22.2) | ***0.86 (0.78-0.94)*** | 8129 (19.5) | ***0.83 (0.78-0.88)*** |
| Multiple prescribers | 20 (0.04) | 11 (0.1) | 1.47 (0.70-3.09) | ≤10 | 1.39 (0.59-3.30) | 10 (0.02) | 0.58 (0.27-1.25) |
| Missing / unclear | ≤10 | 0 |  | 0 |  | ≤10 |  |
| **Disposable income the year before the 1^st^ BZDR dispensation** |  |  |  |  |  |  |  |
| Lowest quartile | 12474 (28.0) | 4579 (28.2) | 1.00 | 3165 (28.7) | 1.00 | 12411 (29.8) | 1.00 |
| 2^nd^ quartile | 14996 (33.7) | 5790 (35.7) | 1.05 (1.00-1.10) | 3725 (33.8) | 1.01 (0.95-1.06) | 13301 (31.9) | ***0.94 (0.91-0.97)*** |
| 3^rd^ quartile | 9761 (21.9) | 3482 (21.5) | 0.96 (0.91-1.01) | 2340 (21.3) | 1.00 (0.94-1.07) | 8916 (21.4) | 1.03 (0.99-1.08) |
| Highest quartile | 7156 (16.1) | 2339 (14.4) | ***0.87 (0.82-0.92)*** | 1759 (16.0) | 1.03 (0.96-1.10) | 6979 (16.8) | **1.11 (1.06-1.16)** |
| Missing / unclear | 138 (0.3) | 30 (0.2) |  | 25 (0.2) |  | 52 (0.1) |  |

*Note:* Significant estimates are written in bold. Inverse associations are written in Italics. The analyses are conducted among the complete analytical sample, i.e., among the individuals with all data on covariates available. Covariates were removed from the analyses if data in the register were incomplete with a substantial proportion of missing values (i.e., civil status) or if characteristics were not relevant for this age group since individuals are at or above the age of retirement (i.e., unemployment, disability pension and social welfare). If a certain characteristic is reported only in 10 or less individuals, the actual frequencies are replaced with ‘≤10’ to mitigate any possibility for backward identification.

*Abbreviation*: BZDR, benzodiazepines and benzodiazepine-related drugs; CI, confidence intervals; OR, odds ratio
